# Hyper-resolution in X-ray emission spectroscopy: integrating extended-range high energy resolution fluorescence detection and multiple-crystal spectrometry with advanced binary data splicing

**DOI:** 10.1107/S1600577525004084

**Published:** 2025-06-17

**Authors:** Ramesh Rijal, Jack Stephens, Daniel Sier, Nicholas T. T. Tran, Truong V. B. Nguyen, Jonathan W. Dean, Pierce Bowman, Minh Dao, Paul Di Pasquale, Tony Kirk, Chanh Q. Tran, Shusaku Hayama, Matteo Aramini, Nitya Ramanan, Sofia Diaz-Moreno, Christopher T. Chantler

**Affiliations:** a School of Physics University of Melbourne Melbourne Victoria Australia; b Department of Chemistry and Physics La Trobe University Melbourne Victoria Australia; c Diamond Light Source Harwell Science and Innovation Campus DidcotOX11 0DE United Kingdom; University of Essex, United Kingdom

**Keywords:** XR-HERFD, extended-range high energy resolution fluorescence detection, multiple-crystal spectrometers, advanced binary data splicing, manganese

## Abstract

This study of manganese (Mn, *Z* = 25) introduces a novel combination of extended-range high energy resolution fluorescence detection (XR-HERFD), multiple-crystal spectrometers and advanced binary data splicing techniques to address challenges in X-ray emission spectroscopy. XR-HERFD enhances spectral precision by utilizing high-resolution crystal analysers and optimized detector configurations. The systematic application of these methods using multiple Bragg crystal analysers at Diamond Light Source has led to substantial improvements in data quality. Simultaneously, advanced binary data splicing integrates multiple datasets to correct distortions and improve resolution, resulting in sharper spectral features. Our results show a significant increase in peak counts and a notable reduction in full width at half-maximum (FWHM), with peak amplitudes increasing by 83% and resolution improving by 46%. These developments provide greater detail for X-ray absorption or emission spectra, offering valuable insights into complex materials, and permitting advances and breakthroughs in atomic relativistic quantum mechanics, chemical sensitivity of atomic transitions and modelling of solid-state effects.

## Abstract 2

A robust integration is introduced of the extended-range high energy resolution fluorescence detection technique, multiple-crystal spectrometers and binary data splicing techniques for the further refinement of spectra in X-ray emission spectroscopy, revealing deeper insights into material properties and atomic transitions.

## 1. Introduction

X-ray emission spectroscopy (XES) is a fundamental technique in analytical chemistry and materials science, offering critical insights into the electronic structure and chemical environment of materials (Nanda & Anjaneyulu, 2017 ▸; Roussel *et al.*, 2018 ▸; Bianchini *et al.*, 2020 ▸; Kroll *et al.*, 2021 ▸). XES has been much improved in the past few decades, particularly in enhancing spectral resolution, moving from early X-ray-sensitive photographic emulsions and traditional solid-state detection based on silicon or germanium semiconductors (Barkla, 1910 ▸; Moseley, 1913 ▸; Fricke, 1920 ▸; Lindsay, 1931 ▸; Jaklevic *et al.*, 1977 ▸) to higher energy resolution crystal analysers and transition-edge sensors (Sparks, 1974 ▸; Eisenberger *et al.*, 1976 ▸; Hämäläinen *et al.*, 1991 ▸; de Groot, 2001 ▸; de Groot *et al.*, 2002 ▸; Gallo & Glatzel, 2014 ▸; Vacher *et al.*, 2020 ▸). These improvements in resolution are crucial for probing complex systems and increasing the precision of structural and electronic analysis.

One of the notable advancements in recent years is the development of high energy resolution fluorescence detection (HERFD) (Glatzel & Bergmann, 2005 ▸; Glatzel *et al.*, 2007 ▸; Sokaras *et al.*, 2013 ▸) and its extended variant, XR-HERFD (extended-range HERFD). These techniques enhance the resolution of X-ray absorption fine structure (XAFS) spectra collected in fluorescence detection mode, allowing for detailed investigations of resonant transitions and satellite processes. XR-HERFD, developed from HERFD and resonant inelastic X-ray scattering (RIXS), has revealed various new physical processes in transition metals, including manganese (Tran *et al.*, 2023 ▸; Sier *et al.*, 2024 ▸). HERFD minimizes the effect of lifetime broadening on the achievable energy resolution of the absorption spectrum, thus providing clearer insights into electronic structure (Kobayashi *et al.*, 2022 ▸). Whilst XR-HERFD offers significant advances in spectroscopic analysis, it is essential to consider its limitations, such as the need for sophisticated instrumentation and expertise, which may restrict its accessibility in some research settings. The design of the I20-Scanning beamline at Diamond Light Source (DLS) has been optimized for this technique and has yielded robust results (Hayama *et al.*, 2021 ▸; Tran *et al.*, 2023 ▸; Sier *et al.*, 2024 ▸). The improvements in instrumentation have enabled XES measurements with an increase in resolution of perhaps two orders of magnitude, with consequent potential added insight. Similarly, HERFD-XAS can provide two orders of magnitude higher resolution and insight over conventional X-ray absorption spectroscopy (XAS), but it is also important to note that these can measure different physical objects. One detail relates to recommendations of crystallographers for many decades that, if one wishes to measure characteristic radiation, *i.e.* XES, then the excitation energy of the incident X-ray should be several times that of the relevant edge energy.

Most high energy resolution detection XES spectrometers at synchrotron beamlines use spherical, Rowland circle or segmented spherical geometry and Bragg bent crystals to achieve high energy resolution and efficient photon collection (Hazemann *et al.*, 2009 ▸; Duan *et al.*, 2017 ▸; Moretti Sala *et al.*, 2018 ▸; Glatzel *et al.*, 2021 ▸; Mei *et al.*, 2024 ▸). Point-to-point focusing spectrometers generally employ Johann geometry (Huotari *et al.*, 2005 ▸; Kavčič *et al.*, 2012 ▸; Sokaras *et al.*, 2013 ▸; Kvashnina & Scheinost, 2016 ▸), while dispersive spectrometers are based on von Hamos geometry (Hayashi *et al.*, 2004 ▸; Huotari *et al.*, 2006 ▸).

High-accuracy fundamental experiments have used Johann Rowland circle geometries to attain energy calibration, measurement and accuracy to approximately 2 parts per million in the X-ray regime or down to *ca* 16 meV resolution from 4 keV to above 16 keV (Silver *et al.*, 1987 ▸; Laming *et al.*, 1988 ▸; Chantler *et al.*, 2000 ▸; Chantler *et al.*, 2006 ▸; Chantler *et al.*, 2007 ▸; Chantler *et al.*, 2009 ▸; Chantler *et al.*, 2012 ▸; Chantler *et al.*, 2014 ▸; Dean *et al.*, 2019 ▸; Dean *et al.*, 2020 ▸). Flat crystal spectrometers have also been used with similar resolution, though usually without calibration reference lines.

Scanning instruments can present intriguing mechanical challenges, particularly when multiple analyser crystals are involved. Achieving a precise relative displacement between these crystals is crucial for optimal performance (Alonso-Mori *et al.*, 2012 ▸; Llorens *et al.*, 2012 ▸; Sokaras *et al.*, 2013 ▸; Duan *et al.*, 2017 ▸). The pioneering work (Wang *et al.*, 1997 ▸) of implementing multiple-crystal analysers has now been widely adopted across various synchrotrons, with each facility customizing the number of analysers depending on beamline designs, funding and specific requirements (Verbeni *et al.*, 2009 ▸; Hazemann *et al.*, 2009 ▸; Duan *et al.*, 2017 ▸; Moretti Sala *et al.*, 2018 ▸; Glatzel *et al.*, 2021 ▸). For a typical beamline, the resolution when performing high energy resolution detection is not Bragg limited, but is commonly a fraction of an electronvolt, so perhaps 1000 or 2000 parts per million. This report develops the evolution of multiple-crystal analysers for the I20-Scanning beamline at Diamond Light Source, emphasizing capabilities, improvements and stability.

In parallel with the developments in XR-HERFD and the precision of multiple-crystal analysers, the sophisticated data processing technique known as binary data splicing can further refine the spectra (Sier *et al.*, 2025 ▸). This technique includes integrating multiple data sets to improve signal-to-noise ratios and achieve higher resolution. It facilitates the characterization and removal of unwanted systematics from measurements and aids in identifying contamination in the sample or beamline path. The rationale, methodology of implementation and results are discussed in Section 3.

The fusion of these advances marks a significant leap forward in X-ray emission spectroscopy. This work examines the integration of these developments, emphasizing their role in elevating and refining the field. In this paper, we will review the principles behind these methods and present developments, and discuss their impact on research and future applications.

## 2. Experimental setup

**Figure 1:**
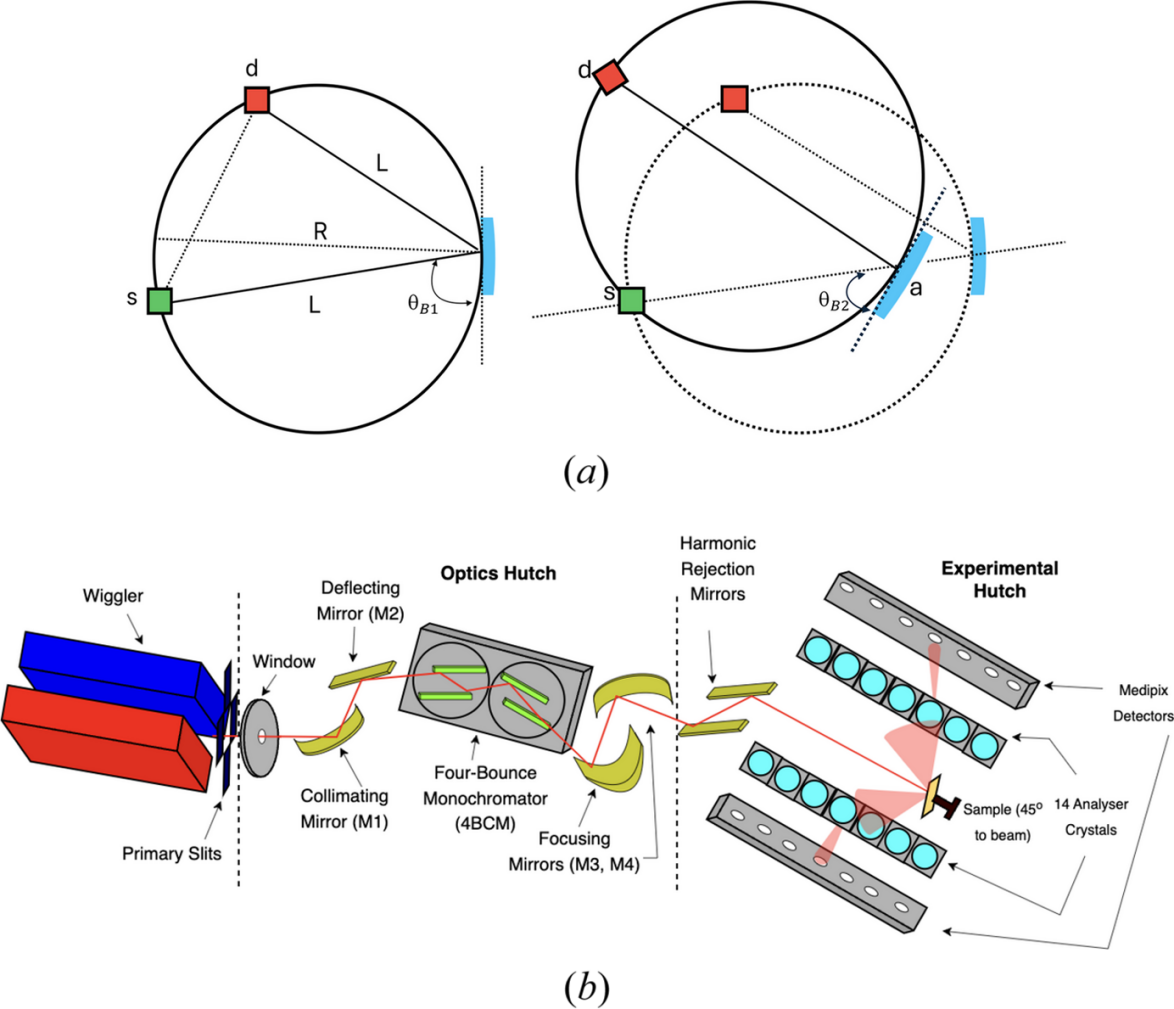
(*a*) Rowland geometry experimental setup. A diagrammatic representation of the Rowland circle geometry is shown. The photon source (s), detector (d) and spherically bent analyser crystal (a) are positioned on a circle with a radius equal to half the bending radius (*R*) of the analyser crystal. The Bragg angle (θ_B_) is defined between the source and the analyser and can be adjusted by moving the analyser and detector along the Rowland circle while keeping the source fixed. Each of the three or 14 analyser crystals, along with their corresponding positions on the detector, is arranged on a separate Rowland circle that intersects at the common source point. (*b*) Schematic of the experimental setup, showing the new 14-analyser spectrometer of the I20-Scanning beamline at Diamond Light Source. The X-rays produced by the wiggler are first vertically collimated, then monochromated using a sophisticated four-bounce monochromator, ensuring high spectral purity. Subsequently, precision focusing mirrors are employed to achieve optimal horizontal and vertical beam focusing, enhancing spatial resolution and intensity for high-resolution experiments.

All the data analysed here were collected on the I20-Scanning beamline at DLS between 2021 and 2024 (Diaz-Moreno *et al.*, 2009 ▸; Diaz-Moreno *et al.*, 2018 ▸). This beamline utilizes a 2 m wiggler and is equipped with a custom-built four-bounce monochromator featuring two pairs of counter-rotating crystals arranged in a (+ − − +) configuration [Fig. 1 ▸(*b*)]. The monochromator is equipped with Si(111) crystals to cover the energy range from 4 to 30 keV, with the crystals cryogenically cooled to handle the heat load from the X-ray source. As a result, the system offers high stability and reproducibility, maintains a fixed exit geometry and ensures that the energy resolution remains independent of the incident beam’s divergence (Hayama *et al.*, 2018 ▸). One of the key strengths of I20-Scanning in conventional XAS mode is its ability to analyse the local structure around a photoabsorbing atom, even when it is present in low concentrations in solution or situated within a challenging matrix. This is due to the beamline’s high flux and spectral purity. These are critical design features. The incident photon flux is high, exceeding 10^12^ photons per second when utilizing the Si(111) monochromator (Diaz-Moreno *et al.*, 2018 ▸).

Most X-ray emission spectrometers at various synchrotron facilities utilize multiple bent crystal analysers operating under Bragg scattering conditions, often close to the backscattering angle (Hazemann *et al.*, 2009 ▸; Duan *et al.*, 2017 ▸; Moretti Sala *et al.*, 2018 ▸; Glatzel *et al.*, 2021 ▸). Likewise, the I20-Scanning spectrometer employs a Rowland circle with a fixed diameter of 1 m based on Johann-type geometry operating in the vertical plane. This geometry employs spherical or cylindrical analyser crystals bent to a radius *R* and the detectors are positioned along a Rowland circle with a diameter of *R* [see Fig. 1 ▸(*a*)]. In this configuration, the analyser crystal(s) collect and focus photons emitted from the sample onto the detector via symmetric Bragg reflection. Employing a vertical Rowland geometry effectively eliminates sensitivity to sample orientation. This design maximizes the photon capture efficiency of the spectrometer and provides an energy resolution of the order of 1 eV, which is sufficient to address core–hole lifetime broadening effects at the *K* edges of the sample. In 2021, the system was equipped with three crystal analysers. However, in 2023 it underwent a significant upgrade, replacing the earlier setup with fourteen crystal analysers. This is now fully operational.

The beamline optical layout includes several advanced components: an upwards-deflecting vertical collimating mirror with rhodium and platinum stripes that maximizes flux through the monochromator, followed by a downwards-deflecting vertical mirror that restores the horizontal trajectory of the beam and isolates mirror adjustments, are placed in front of the monochromator. After the monochromator, vertical and horizontal focusing mirrors, both with rhodium and platinum coatings, focus the beam at the sample position. Already located in the experimental hutch, a set of harmonic rejection mirrors with rhodium and silicon stripes maintain high spectral purity by reducing the harmonic content.

### 2.1. Elastic scan

An elastic scan measures elastically scattered photons, distinct from the emission (fluorescence) from the sample, and should not be confused with the latter. The purpose of performing an elastic scan is to determine the spectrometer’s energy resolution experimentally without the need to account for the natural linewidth of the emission line. To do this, the scan is carried out at an energy close to the emission line of interest but below the excitation threshold of the material, ensuring no fluorescence is generated. This measurement is typically performed using a scattering material, though in some cases, for convenience, the sample itself may be used.

### 2.2. Detector configuration and region of interest optimization

A key step in statistics and information is to improve the solid angle with multiple curved crystal analysers diffracting to detectors. A second key point is to have each analyser crystal signal proceeding to a different or separate section of the detector. Whilst the technique of deliberately separating each of the analyser crystals on the detectors is not entirely new (Hayashi *et al.*, 2004 ▸), problems in alignment and processing have led to this being non-standard and rarely used on most such beamlines. Some have used one point detector per analyser crystal, but without the potential to define a region of interest (RoI) to deal with background signals. Conversely, the setup designed on the I20-Scanning beamline has demonstrated significant improvements in both spectral intensity and resolution over the past few years. Additionally, this approach addresses several issues that can arise when analyser images overlap, such as motor misalignments or alignment faults (Sier *et al.*, 2024 ▸).

In general, the use of a two-dimensional detector enhances the potential for routine and advanced analysis. These experiments utilized a MAXIPIX (multichip area X-ray detector based on a photon-counting pixel array) TAA22PC detector, a development of the Medipix2 single-photon counting pixel detector (Ponchut *et al.*, 2011 ▸). We refer to these MAXIPIX detectors based on silicon sensors as Medipix. They are configured in a 4 × 1 arrangement, creating a total pixel grid of 1024 × 256 pixels. The original instrument was equipped with one detector that collected the images of the three analyser crystals. Since the upgrade, two detectors are used, each collecting the images from seven crystal analysers, thereby improving both the spatial resolution and the overall sensitivity of the measurements. The Mn foil sample, obtained from Goodfellows, had a stated purity of 98.7% and measured 25 µm × 25 µm in area. The beam size at the sample was 400 µm horizontally and 300 µm vertically, offering a well focused probe for experimental analysis. For the low-temperature series, the sample was mounted in a cryostat using liquid nitrogen and helium, successfully achieving temperatures as low as 10 K during the experiment.

**Figure 2:**
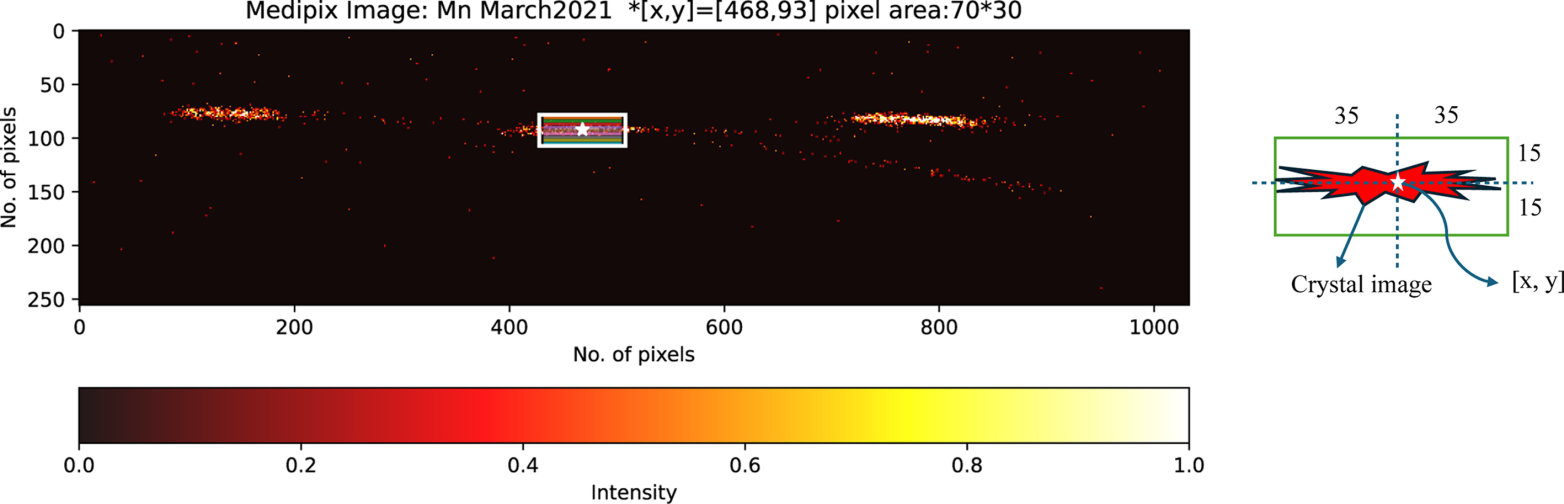
Image displaying reflections of the three Ge analyser crystals on the Medipix detector. The central crystal is highlighted by a white rectangular region of interest (ROI). A schematic diagram is on the right, showing a single crystal image (in red) and explaining the ROI selection process, with all dimensions in pixels. The white star marks the central coordinate (*x*, *y*) of the central analyser. The size and position of the ROI can be adjusted to give the best match to the focused image of the crystal on the detector.

Another key point is that the pixel images of each analyser should be well separated to minimize overlap and ensure a detailed isolated image of each analyser crystal. To find the precise RoI, a central coordinate (*x*, *y*) is chosen from each of the crystal analysers (Fig. 2 ▸). A rectangular segment, highlighted in white, extends *e.g.* 35 pixels on either side of this coordinate along the horizontal axis and 15 pixels above and below along the vertical axis, making the RoI for each analyser crystal a 70 × 30 rectangular region. The *Data Analysis Workbench* (*DAWN*) software can read the raw output files and be used to locate the precise central coordinate (Basham *et al.*, 2015 ▸).

## 3. Experimental and analytical data splicing to improve statistics and resolution for a single-crystal analyser diffraction image

Experimentally, there are numerous engineering methods for attaining HERFD with diffracting analyser crystals. In all cases, selecting a diffracting crystal type where the diffraction is near back-reflection increases the resolution and decreases defect broadening, whether from geometric or strain distortions. Selecting a ‘diced’ or ‘sliced’ crystal rather than a monolithic spherical crystal can significantly reduce focusing defects. Selecting an ideal cylindrical curved crystal in Johann mounting is arguably the most feasible way to optimize both resolution and flux and to minimize geometric and strain defects. Selecting a perfect flat crystal can improve resolution and minimize particular strain defects; but it also limits the flux, statistics and solid angle dramatically, and introduces significant geometric defects and calibration limitations. In this study, we focus on cylindrically sliced crystals, where the crystal is cut into thin cylindrical shapes and mounted on a spherical template. This configuration follows a Johann mounting, optimizing the solid angle, statistics and resolution. We attempt to answer the question of how we can, experimentally and analytically, measure and improve the resulting resolution without loss of statistical accuracy, or with minimal loss of statistics, especially using data splicing.

**Figure 3:**
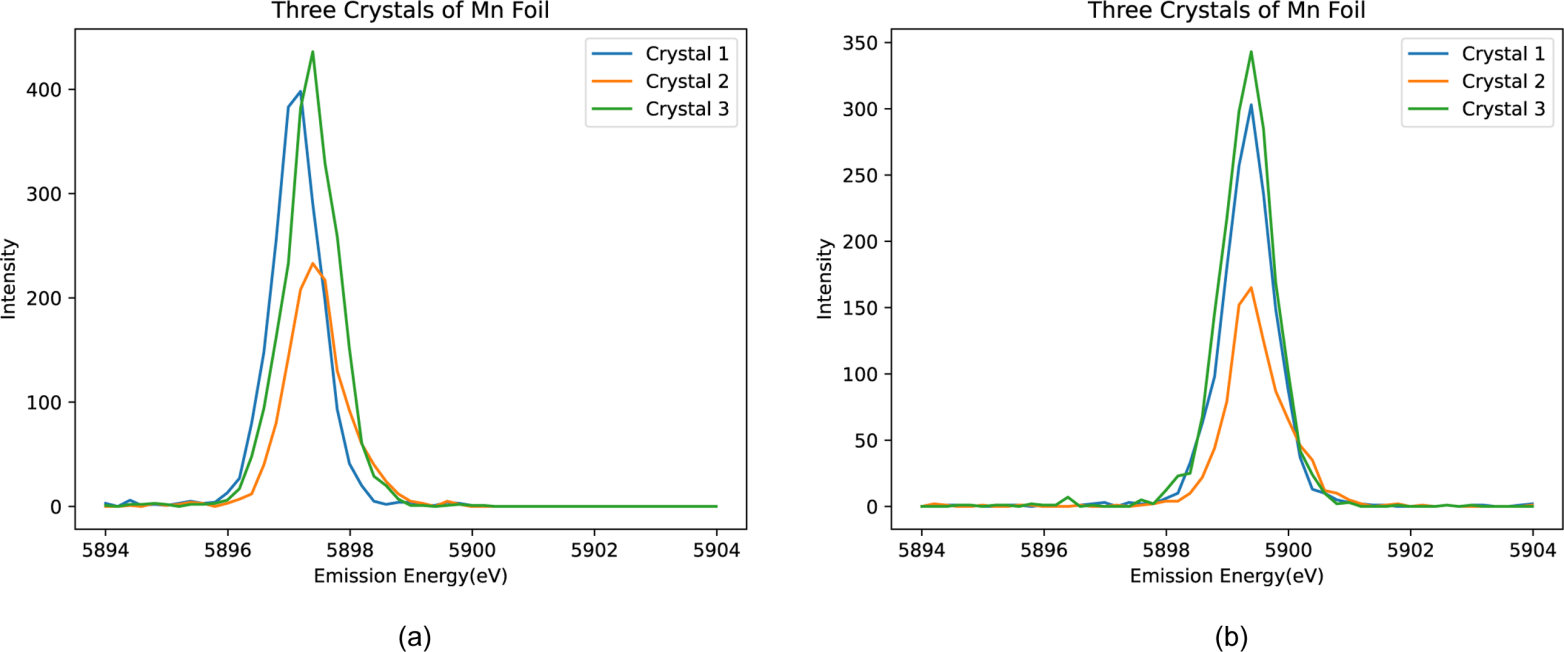
Three elastic spectra of Mn foil generated from the three-crystal analyser from (*a*) March 2021 (Experiment 1) and (*b*) December 2021 (Experiment 2) at incident energy 5898.6 eV. There is an alignment shift of up to 0.4 eV on the emission axis between the crystal analysers in Experiment 1, the first time these images were separated on this beamline, which is corrected in Experiment 2. The FWHM value for both experiments is approximately 1 eV.

The first such separated crystal experiment at Diamond used a spectrometer with three crystals (Fig. 3 ▸) in March 2021 (henceforth called Experiment 1). Significant peak distortions are common, here and elsewhere, across almost all of the slices, which stem from various sources. Instrumental factors, such as misalignment, calibration issues, or non-uniform curvature and offsets in the detector image, can significantly impact data accuracy. Additionally, the quality of the sample, including defects or strains in the crystal, can affect peak shapes. Variations in experimental conditions, such as temperature or pressure, may also contribute to these distortions, and inaccuracies in data processing or analysis algorithms can introduce artefacts. The spectrometer energy resolution (including how close the setup is to backscatter), the nature of the curvature, and stress and strain in the analysers, all help to define the observed resolution, affected by beam divergence, bandwidth, size of the beam on the sample and penetration depth. If both the incident bandwidth on the sample and the sample scattering were ideal (Dirac δ-functions), crystal diffraction would closely reflect the intrinsic Bragg width. By conducting an elastic scan on XES spectra, one can identify the resolution and limitations.

**Figure 4:**
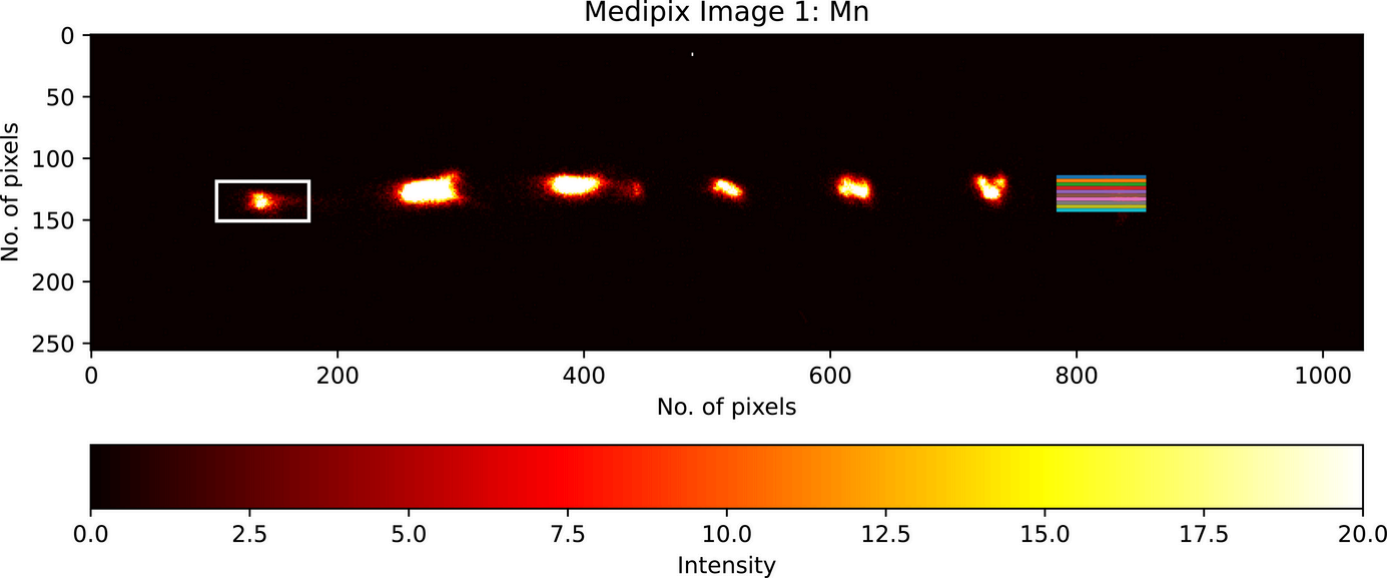
Representative Medipix image of manganese foil at an incident energy of 6490.4 eV, featuring the seven crystal analyser images on the first detector (Experiment 4). The first crystal image, highlighted by a white rectangle (70 × 30 pixels), represents our selected RoI and is referred to as raw or ‘unspliced’ data. The final or seventh crystal image illustrates the ten bands or slices (each 70 × 3 pixels) selected for splicing, optimized based upon the image and geometry.

Honkanen *et al.* (2014 ▸) introduced a model that analyses the spectrum from the analyser surface by dividing it into a fine grid, calculating centroid energy shifts and generating ‘virtual photon counts’ to achieve statistical accuracy consistent with experimental data. In a similar vein, we refined our energy resolution by partially mitigating the broadening intrinsic to the source, sample and divergence, on the Johann geometry, exploiting the spatial resolution provided by the Medipix in our datasets. To do this, a systematic approach is employed to correct the observed spectral distortions. Firstly, ten narrow horizontal slices are taken across a given crystal, as seen in Figs. 4 ▸ and 5 ▸. The isolated emission spectrum from each slice is then adjusted in energy to align with the true centre of the spectrum, which is determined by fitting a Gaussian profile to the summed spectra over the entire crystal area. Given the orientation of the detection apparatus on I20, it is anticipated that the most significant distortions will occur along the dispersion direction (*i.e.* vertically). Our result demonstrates that this method of realigning the slices effectively reduces spectral broadening and enhances intensity values, resulting in notably sharper peaks.

**Figure 6:**
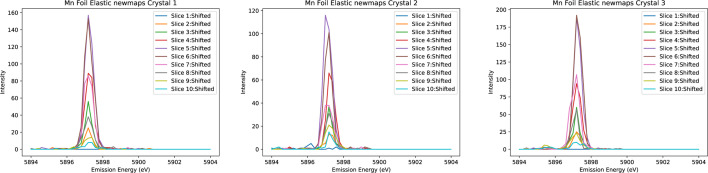
Elastic scans analogous to Fig. 5 but with peaks aligned to the reference spectrum.

**Figure 7:**
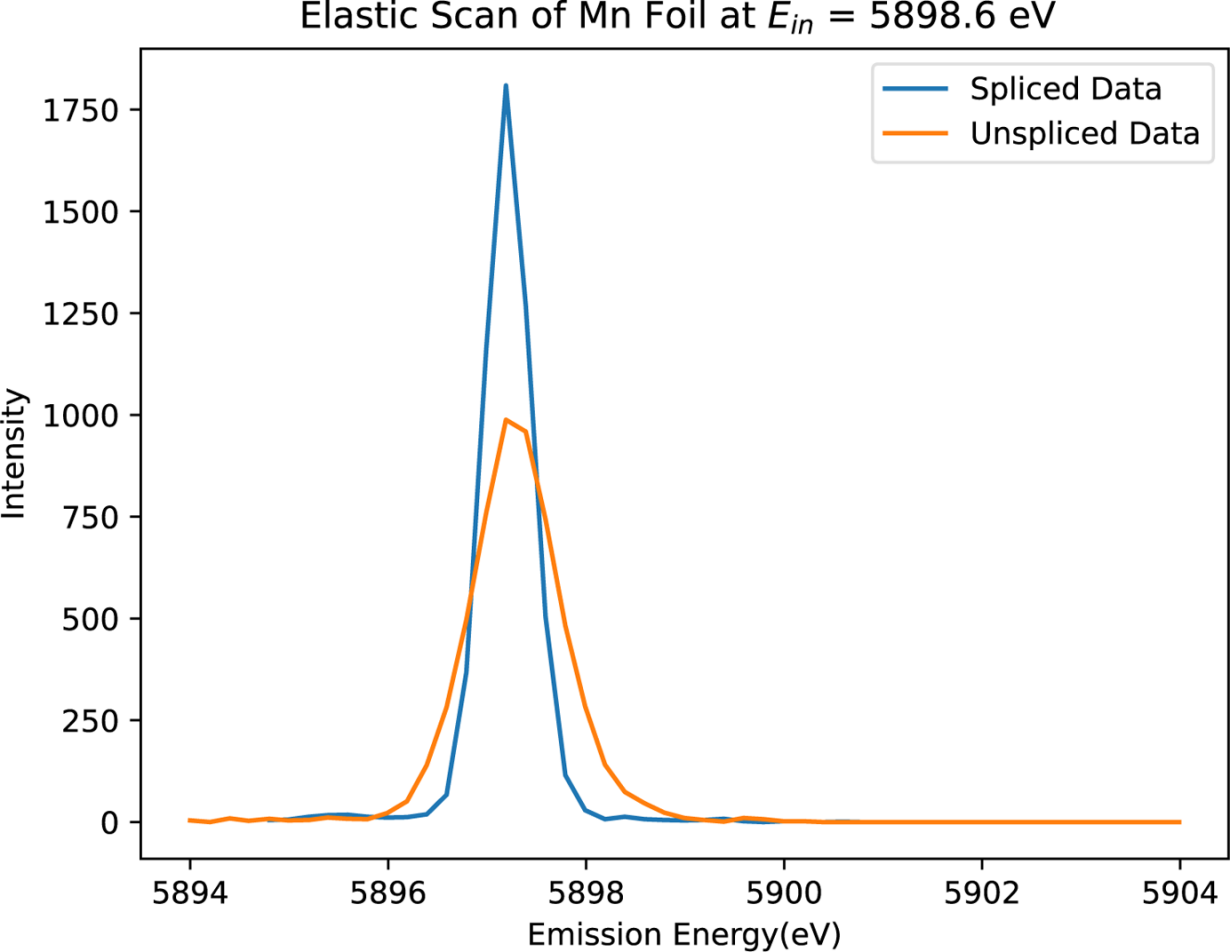
Spliced (processed, blue) versus unspliced (raw, orange) spectra of Mn foil using the three-crystal analyser from Experiment 1. The spliced spectrum demonstrates significant improvement in both peak count and resolution, highlighting the effectiveness of spectral splicing in optimizing data quality and providing a clearer representation of Mn spectra.

**Figure 8:**
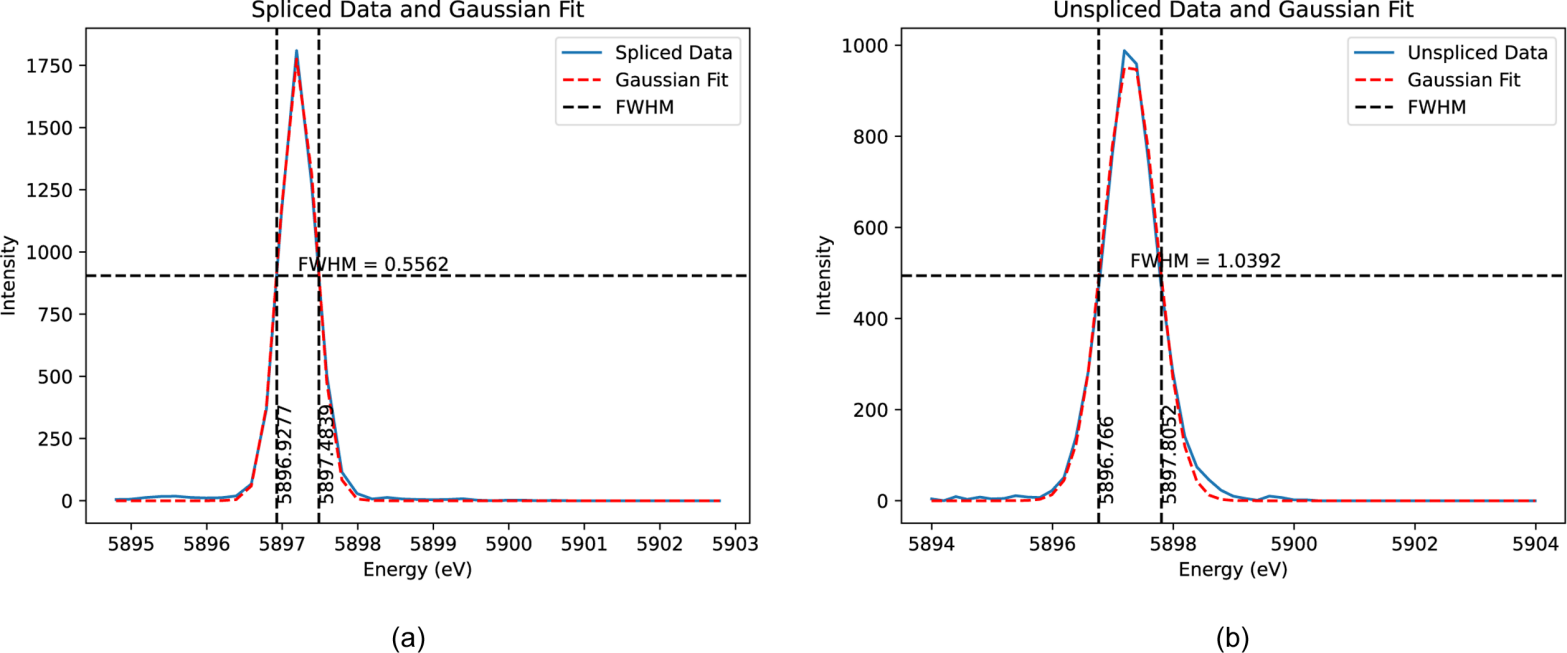
Analysis of FWHM using Gaussian fitting for (*a*) spliced and (*b*) unspliced datasets. The red dashed lines represent the Gaussian fit, accurately modelling the data. The black dashed lines denote the reference energy points used to determine the FWHM values. This method gives a good fit to the observed structure.

After aligning each spectrum to the energy reference (Fig. 6 ▸), the ‘spliced data’ are constructed by aggregating the ten slices. This aggregation significantly enhances both the resolution and peak intensity of the spectrum. Fig. 7 ▸ illustrates a comparison between the unspliced (raw) data and the spliced data, demonstrating the improvements achieved through this process. To determine the accurate FWHM of the spectrum, we employed various methods, with Gaussian fitting emerging as the most effective approach. The calculations derived from this method yielded consistent and reliable results, as shown in Fig. 8 ▸. In our preliminary experiment conducted in March 2021, we observed a notable reduction in FWHM, decreasing from 1.0392 eV to 0.55 eV, representing an improvement in resolution of approximately 46%. Additionally, the intensity peaks became sharper, leading to a peak count difference of up to 83%. Table 1 ▸ highlights the powerful impact of splicing in refining data resolution and enhancing measurement precision.

**Figure 9:**
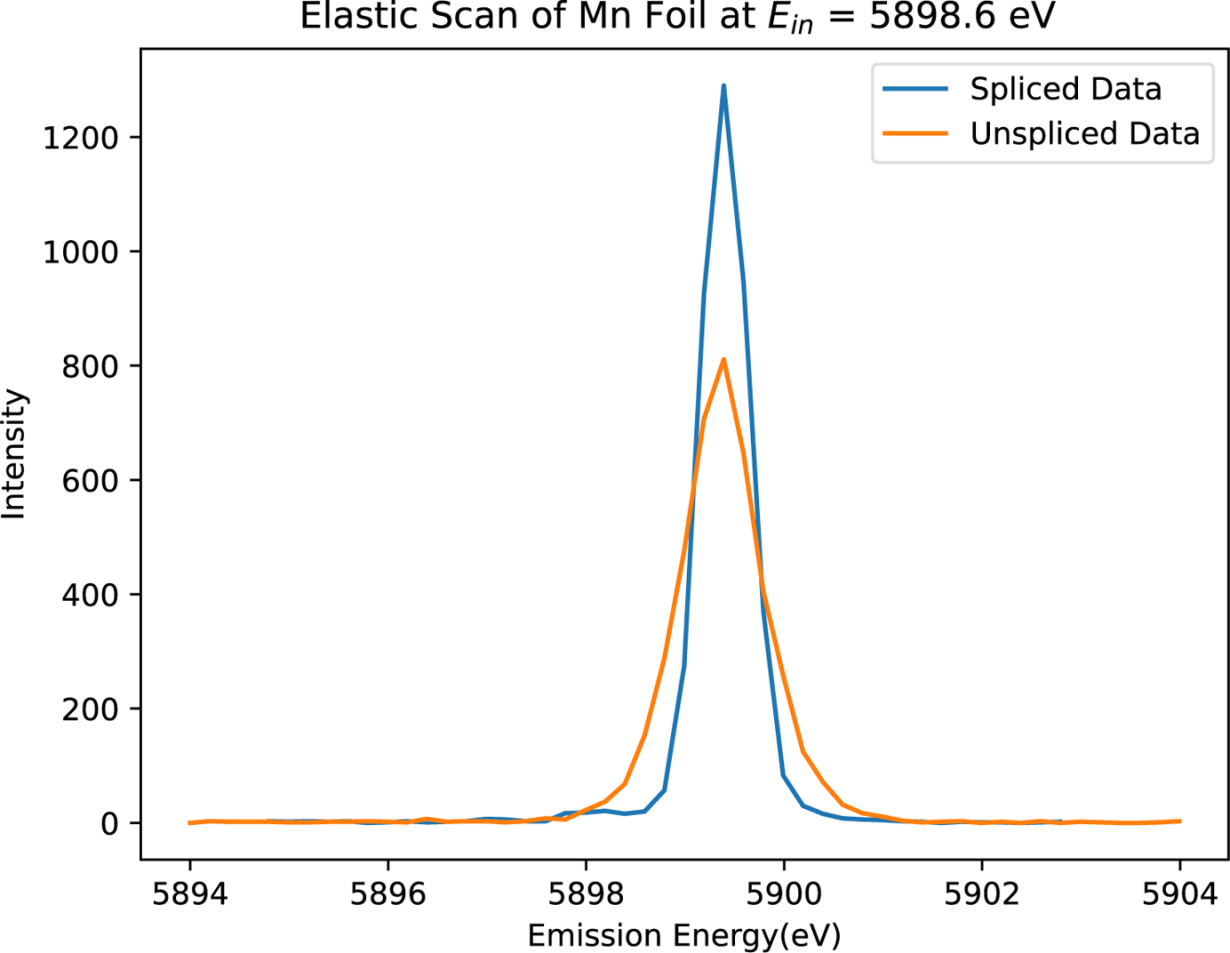
Comparison of spliced (processed, blue) and unspliced (raw, orange) spectra of manganese (Mn) foil obtained with the three-crystal analyser during Experiment 2.

In December 2021, we conducted a second experiment (referred to as Experiment 2) using an Mn foil with a similar crystal setup. In this run, the alignment was improved, particularly for the left-hand crystal, which had been slightly misaligned in the first experiment (Fig. 3 ▸). Splicing the crystal slices as per our standard procedure resulted in the expected exceptional outcomes (Fig. 9 ▸). The FWHM decreased from 1.03 eV in the first experiment to 0.97 eV in the second and further improved to 0.57 eV following the splicing process. The resolution depends on the intrinsic width of the peak, as well as on beamline parameters, including optics, monochromator, spot size and focus on the sample. Resolution and broadening are energy dependent.

## 4. Experimental and analytical details: introducing the 14-crystal spectrometer

**Figure 10:**
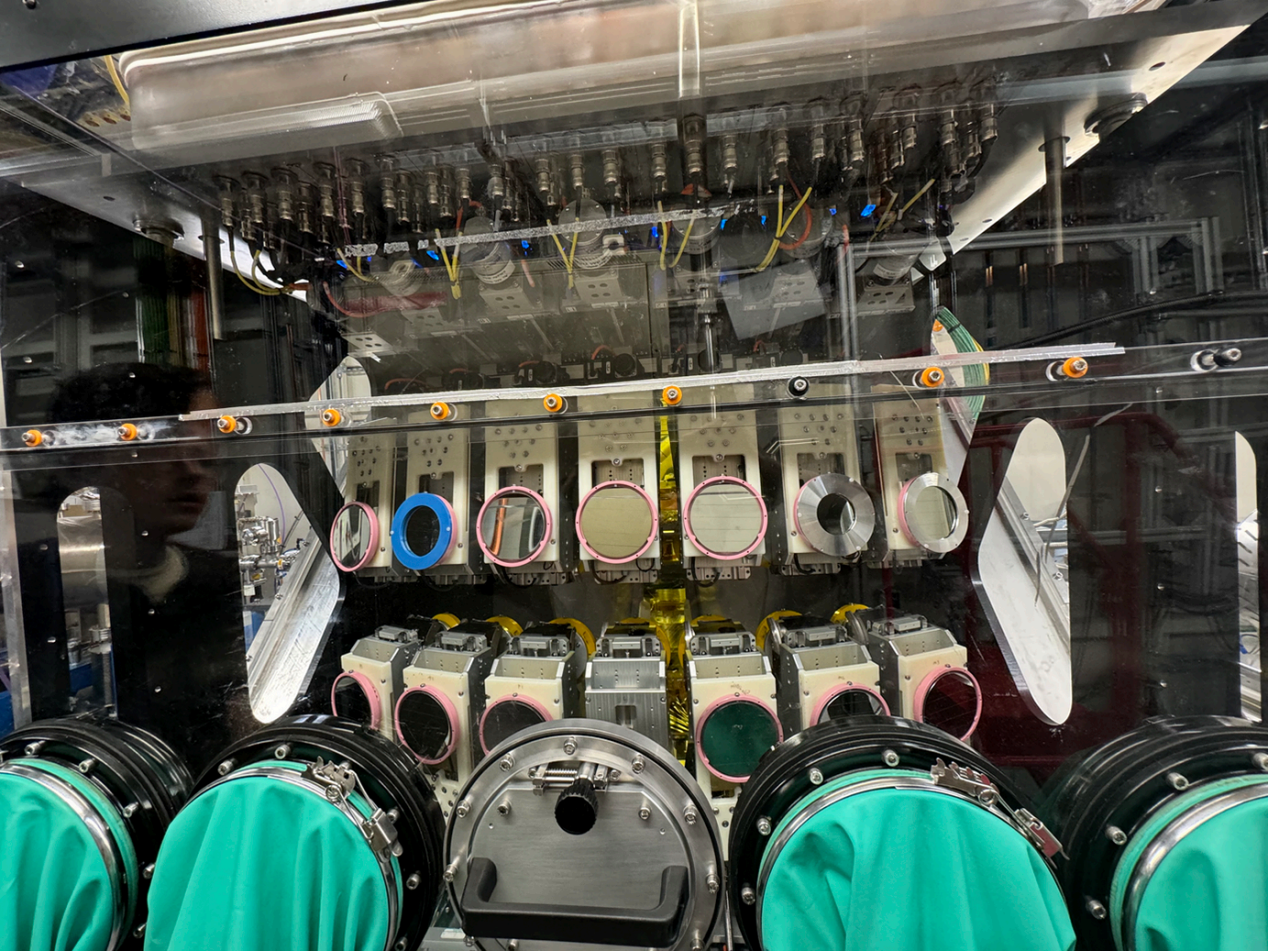
The 14-crystal spectrometer on I20-Scanning for Experiment 4. It includes two separate rows that can be installed with either the same type of crystal to measure a single emission line or different types of crystals to measure distinct edges.

**Figure 11:**
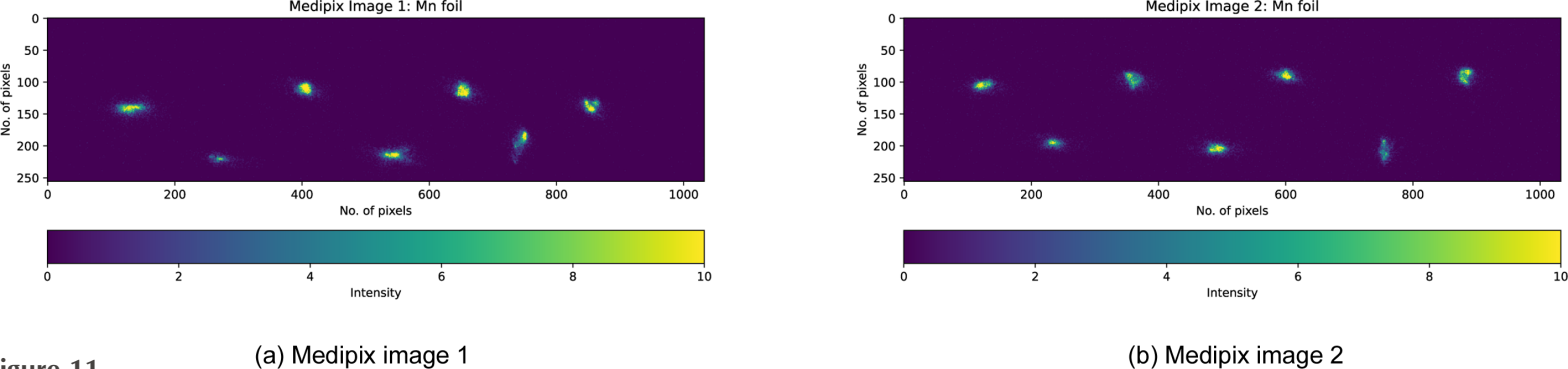
Medipix images of Mn foil at low temperature (Experiment 3) illustrating the ‘4, 3’ arrangement of the 14-crystal analyser setup in two detectors.

In April 2023, the I20-Scanning beamline at DLS was upgraded to a 14-crystal spectrometer featuring two rows of crystals (in two Medipix detectors), each capable of housing either identical crystals for measuring a single emission line or different types to analyse multiple lines simultaneously. The 14-crystal configuration as shown in Fig. 10 ▸ enhances solid-angle and spectral statistics, improving signal-to-noise ratios. This upgrade allows for detailed analysis of complex materials. Experiment 3 arranged the seven crystal images detected by each Medipix into two rows, one containing four images and the other containing three (Figs. 11 ▸ and 12 ▸). As this was the first time such an experiment was conducted on I20, the energy calibration was not perfectly adjusted and some analyser crystals were poorly focused. These limitations highlighted the need for improvement, which motivated the refinements implemented in the subsequent experiment described below.

**Figure 13:**
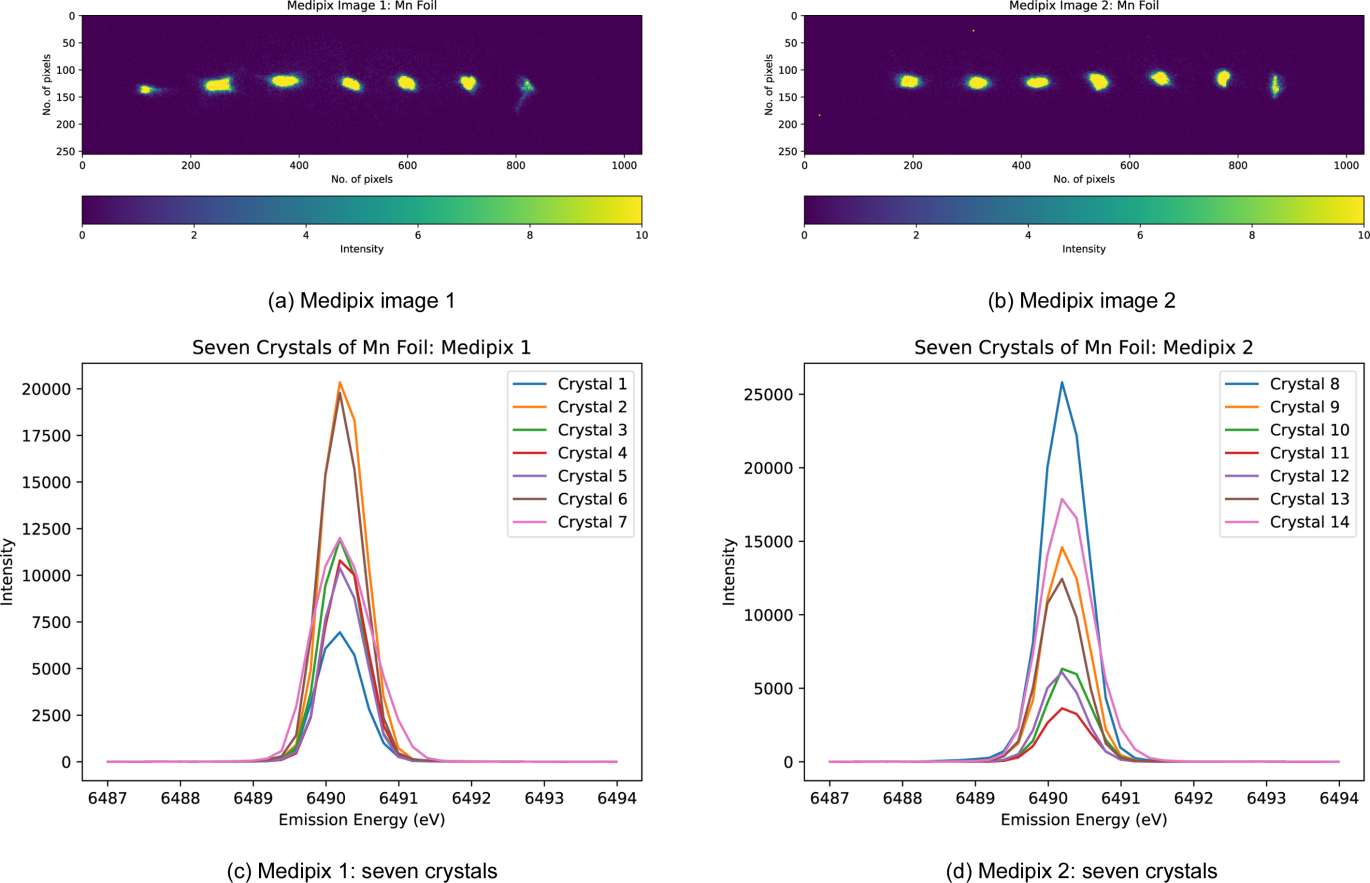
Two Medipix detector images of Mn foil at low temperature (LT = 11 K) (Experiment 4), aligned in the same horizontal space. The normal elastic scan of each individual analyser crystal was performed at the maximum of the Mn *K*β emission peak using the Si(440) reflection with the spectrometer at 84.2°. The crystal images are well aligned, achieving an accuracy of 0.1 eV.

In July 2024 (Experiment 4), I20 successfully aligned the seven crystals in each Medipix within a single horizontal plane. Fig. 13 ▸ illustrates the performance of the new 14-crystal setup for a manganese foil at 11 K. The Medipix images demonstrate improved alignment across the analyser crystals. The adoption of this spectrometer design has led to significant enhancements in count rates and a resolution accuracy up to 0.1 eV, showing the potential to deliver high-quality spectral data.

**Figure 14:**
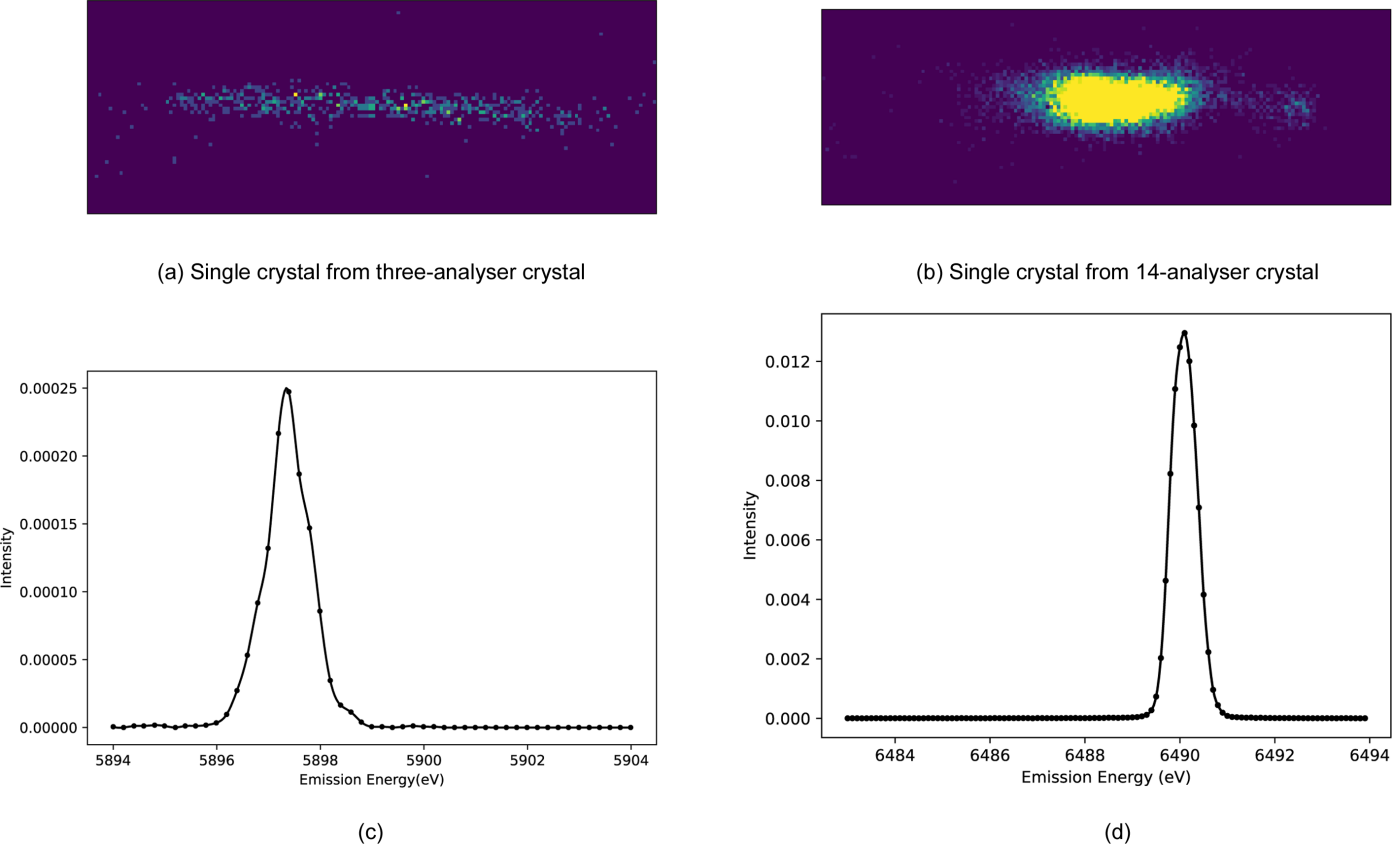
Elastic scans of Mn foil with (*a*) and (*c*) the three-crystal analyser with the Ge(333) reflection set at 74.8° for the *K*α emission line, and (*b*) and (*d*) the 14-crystal analyser with the Si(440) reflection set at 84.2° for the *K*β emission line. Four-bounce Si(111) monochromation was used. The focus is notably sharper in panel (*d*) (the 14-analyser crystal) than in panel (*c*) (the three-analyser crystal). The FWHMs of the elastic peaks are about 1.03 eV and 0.61 eV for (*c*) and (*d*), respectively.

On comparing the spectra from three crystal analysers configured with the Ge(333) spectrometer reflection at 74.8° for *K*α emission lines with the spectra from the 14-crystal analyser with the Si(440) reflection at 84.2° for *K*β emission lines, we noted a significant improvement in focus quality (Fig. 14 ▸), with spectral broadening decreasing from 1.03 eV to 0.70 eV (Table 6).

**Figure 15:**
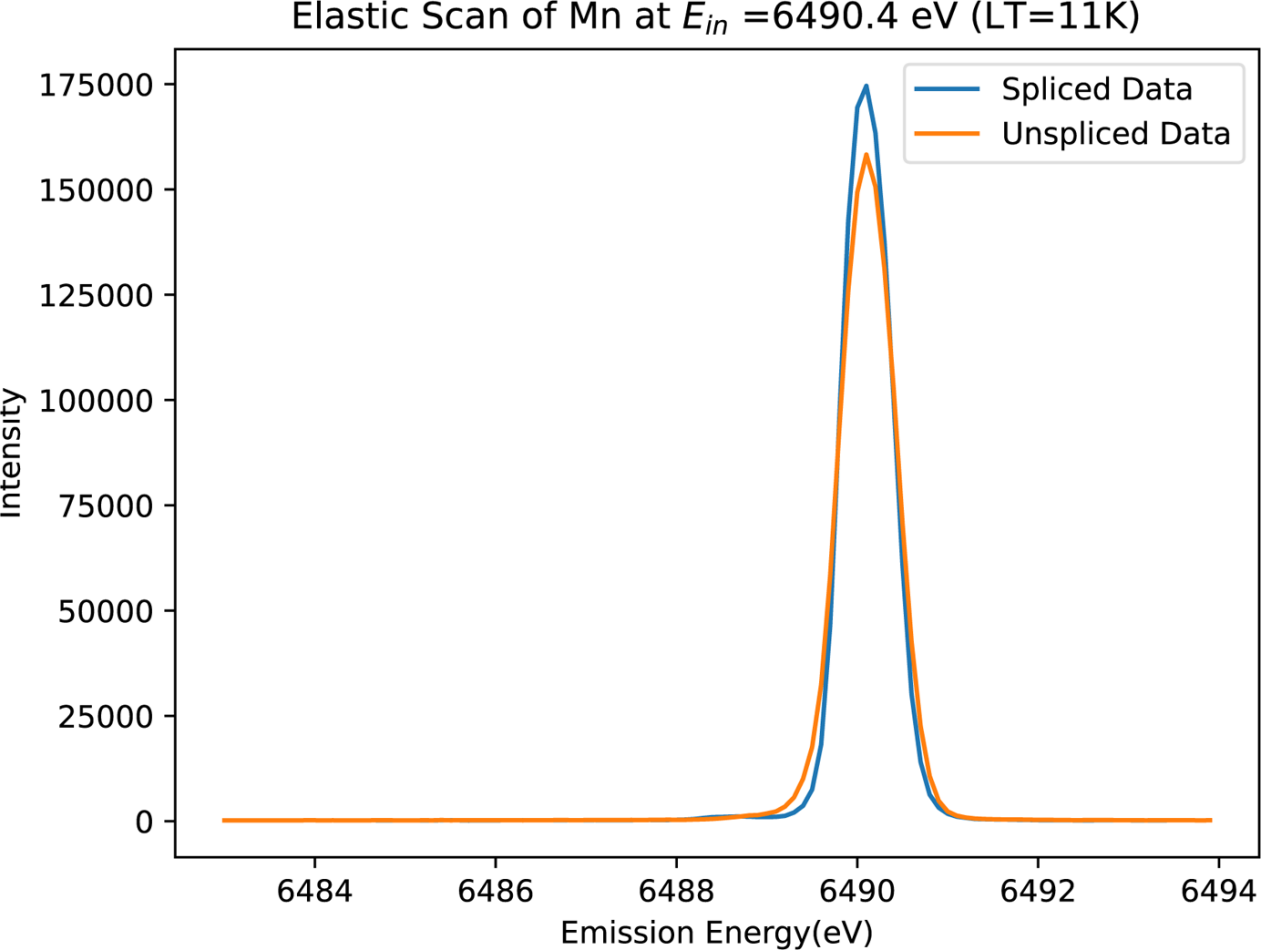
Spliced (processed, blue) and unspliced (raw/normal, orange) spectra of the Mn foil using the 14-crystal analyser (Experiment 4). The spectral broadening has narrowed to 0.70 eV. Resolution and photon counts are increased upon splicing, with these data significantly higher in resolution than earlier experiments.

**Figure 16:**
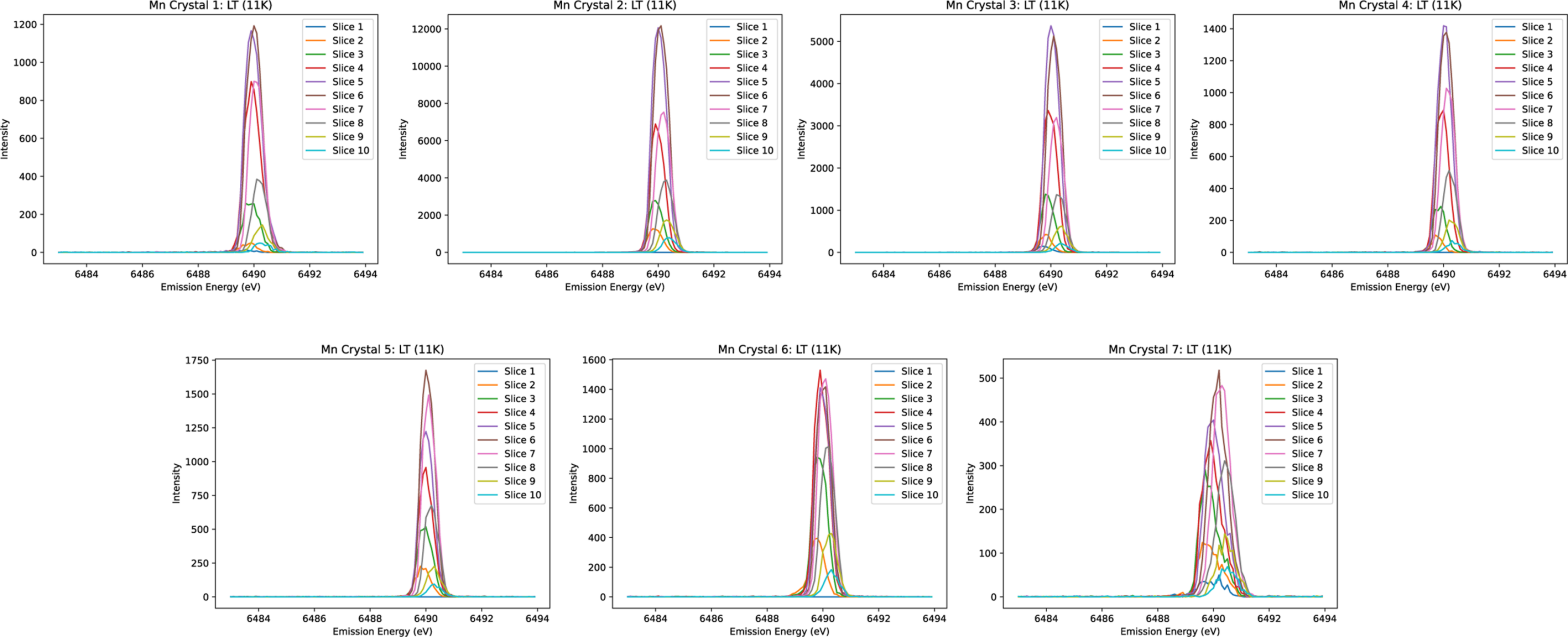
Elastic scans of manganese foil at LT = 11 K from Experiment 4, showing the first seven crystals from the first Medipix detector. Slight shifts of the peaks in each slice can be observed.

**Figure 18:**
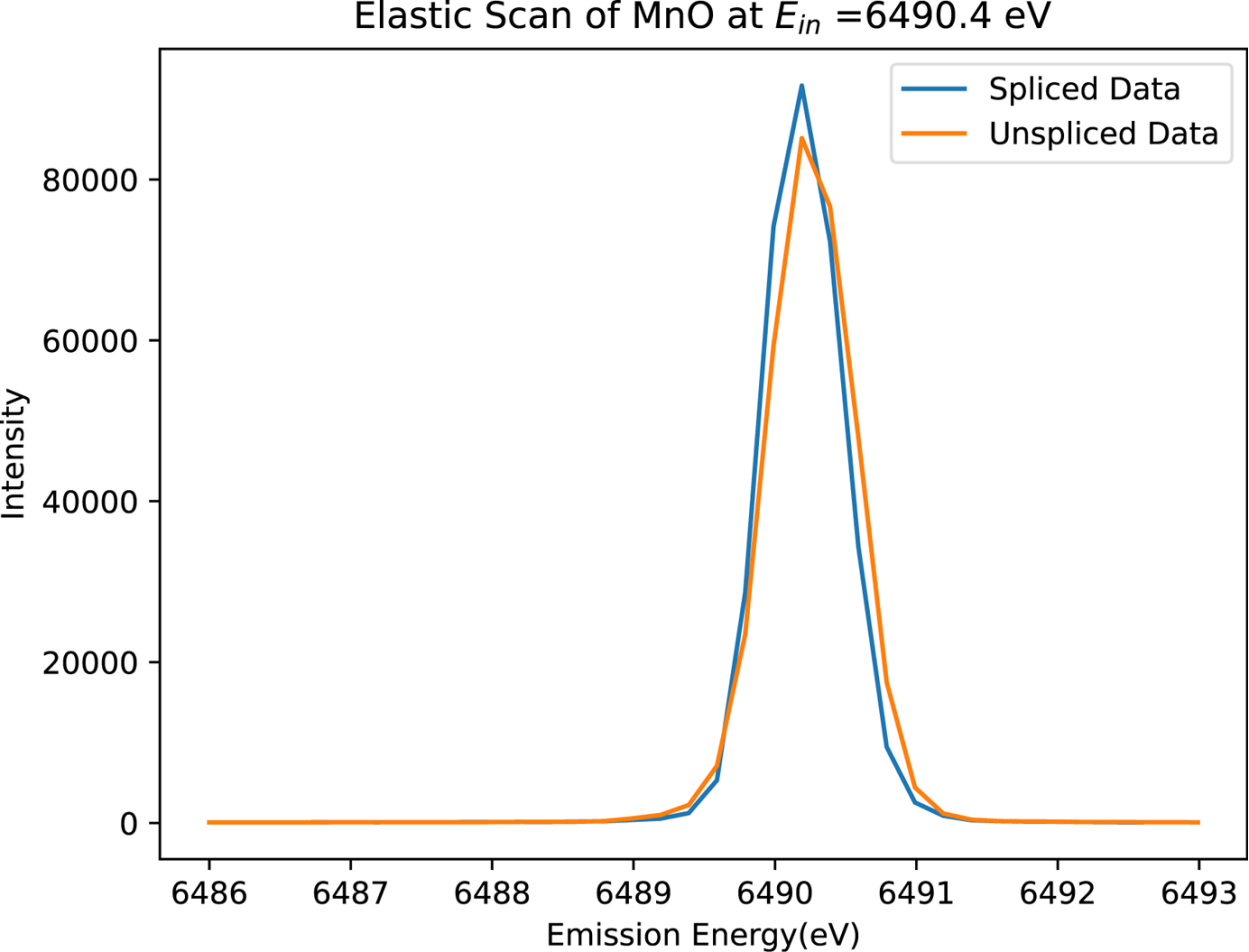
Spliced (blue) and unspliced (orange) spectra of MnO using the 14-crystal analyser (Experiment 4).

Fig. 15 ▸ shows the focused and enhanced spectra of the Mn foil *K*β emission lines with the 14-crystal configuration. In addition, the spliced spectra demonstrate a reduction in spectral broadening of up to 12.2%, along with an increase in signal counts of 10.1% compared with the raw data (Figs. 16 ▸ and 17 ▸). This enhancement confirms the effectiveness of splicing applied to the advanced 14-crystal configuration. Table 2 ▸ summarizes the improvements in spectra using the 14-crystal spectrometer and splicing technique. Further experiments employing a manganese oxide (MnO) sample using the 14-crystal spectrometer showed similar improvements in count rates and FWHM (Fig. 18 ▸).

## 5. Resolution comparisons with theory

The intrinsic broadening observed in optical systems and detectors is well understood. Johann geometry broadening, within the context of the intrinsic ideal Bragg spectrum, is influenced by several factors, including photon depth penetration, finite crystal dynamical diffraction, curved crystal defects, asymmetric diffraction and crystal strain (Chantler, 1992*a* ▸; Chantler, 1992*b* ▸; Chantler, 1995 ▸).

This interplay leads to geometric energy broadening, which affects the image location on the Rowland circle and the detector (Bergmann & Cramer, 1998 ▸; Sutter *et al.*, 2008 ▸). Several approaches have been introduced over the years to address this broadening (Kavčič *et al.*, 2012 ▸).

Spherical crystal broadening is often quite significant (Honkanen *et al.*, 2014 ▸). Following those authors, the error field for an infinitely thin spherical crystal (no thickness strain) is given by 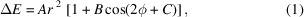
where
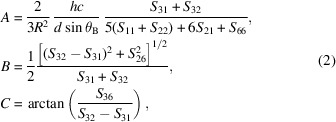
where *r* is the radius of the analyser crystal, ϕ is the angular coordinate (‘between *x* and *r*’), *z* is the axis in the direction of the thickness and *x* presumably relates to the crystal orientation. We emphasize that this excludes the large additional strain and broadening with thickness. Perhaps these are best exemplified in the formulae for spherical bent crystals given by Huotari *et al.* (2017 ▸) under the categories given in Table 3 ▸, with the sample placed a distance 2*z* inside the Rowland circle and the analyser crystal placed a distance *z* outside of the Rowland circle, where *z* ≃ 75 mm or 140 mm.

Note that for more suitable arrangements, the strain aberration can be reduced dramatically: the off-Rowland circle defect can be reduced to approximately zero. The incident bandwidth from the monochromator will definitely add to broadening from the analyser to the crystal but can be corrected for or eliminated following the discussion and experimental results above. Compared with an ideal spherical crystal of large thickness, techniques of dicing (Huotari *et al.*, 2005 ▸), compensating theoretically from image sections of a single spherical crystal (Honkanen *et al.*, 2014 ▸) or chopping into pseudo-cylindrical Johann crystal slices have been used to overcome geometric limitations in broadening, compared with an infinite flat crystal limit.

Typical reference equations for the limiting resolution from the literature include
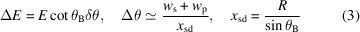
for a perfect cylindrical crystal of infinitesimal thickness, with Rowland circle radius *R*, 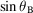 = 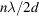 and Δθ the angular divergence of the system, estimated from the (horizontal) beam size on the sample *w*_s_ and the spatial resolution or pixel size of the detector *w*_p_ (Hayashi *et al.*, 2004 ▸). Elastic deformation of the curved Johann-mounted crystal in Rowland circle geometry has distortions and bandwidth estimated as 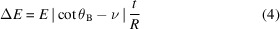
or (Honkanen *et al.*, 2014 ▸; Moretti Sala *et al.*, 2018 ▸) 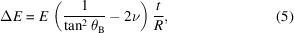
where *t* is the crystal thickness and ν is the Poisson ratio of the analyser crystal material (0.22 for Si and 0.27 for Ge) (Suortti & Freund, 1989 ▸). ‘Dicing’ – that is, chopping the curved crystal in Johann geometry into small cubic pieces to reduce the strain function and distortion – introduces crack defects, but is intended for much higher resolution systems than bent spherical crystals. Diced spherical crystals give an expression like
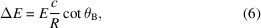
where *c* is the width of the square chopped pieces (Huotari *et al.*, 2005 ▸), or (Verbeni *et al.*, 2005 ▸; Moretti Sala *et al.*, 2018 ▸)
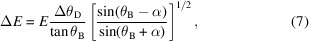
where Δθ_D_ is the Darwin width, which can be defined as: the infinite flat perfect crystal width and formula; the ideal finite flat perfect crystal formula; more complex ray tracing; or proper curved crystal theory. The asymmetric cut angle α is very important, and it changes the angular range incident on the planes and the angular range exiting the crystal surface, and also the angular range and location on the detector (Chantler, 1992*a* ▸; Chantler, 1992*b* ▸; Chantler, 1995 ▸). This may act like a flat crystal, or may be formed like a Johann mount, or may be formed to a cylindrical surface, or in principle may be cut like a Johanssen geometry, which introduces complex diffraction effects whilst removing the strain gradient. It is claimed that the dicing limit can be improved upon by a detector spatial resolution and replaced by a similar function with pixel resolution or pixel width (in the dispersion direction) *w*_p_,
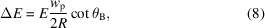
or (Moretti Sala *et al.*, 2018 ▸)
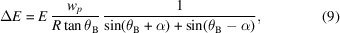
which is usually smaller by an order of magnitude (perhaps *c* ≃ 1 mm, *w*_p_ ≃ 170 µm), where 2*R* is an approximation for the back-scattered *x*_sd_ (Huotari *et al.*, 2005 ▸).

Johanssen geometry and spherical broadening are significantly less well resolved and controlled than Johann geometric cylindrical crystals. There is also a potential Johann aberration from off-axis shifts along the dispersive direction of the analyser (Moretti Sala *et al.*, 2018 ▸; Suortti *et al.*, 1999 ▸), corresponding perhaps for spherical crystals to
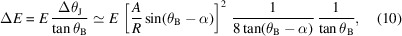
where *A* is the illuminated analyser radius.

A typical contribution summary from the literature is given in Table 4 ▸, noting that some broadening components add (approximately) linearly while others add (approximately) in quadrature or otherwise. These estimates appear to achieve resolution below 0.1 eV, but perhaps at the expense of not including many finite and curved crystal broadening contributions, even for perfect and relatively thin crystals. This sort of resolution is nonetheless achievable for a flat perfect crystal with no strain, but then typically the calibration of position on the detector and from multiple crystals or crystal segments is extremely challenging and the solid angle collection statistic is very poor.

In general, there have been three approaches to the estimation of the width and resolution of crystal monochromators and analysers: a full Takagi–Taupin approach or a Chantler approach, usually with some limitation (Chantler, 1992*a* ▸; Chantler, 1992*b* ▸; Chantler, 1995 ▸), a ray-tracing or X-ray optic (XOP) approach, or as above a selection of relevant components.

The estimated energy resolution of the spectrum consists of both geometric contributions and intrinsic energy resolution. The intrinsic component includes two main factors: the Darwin width, and the broadening resulting from stress developed in the lattice planes during crystal bending (Alonso-Mori *et al.*, 2012 ▸). The Darwin width for a perfect crystal Bragg reflection can be calculated using dynamical diffraction theory (Takagi, 1962 ▸; Taupin, 1964 ▸), with values of 0.065 eV for Si(440) and 0.114 eV for Ge(333) analyser crystals. Geometric contributions stem from the derivative of Bragg’s law and are expected to play a significant role in the overall resolution. Factors such as Johann aberrations, pixel size effects, source size variations and the resolution function of the analyser crystal substantially influence the spectrum’s resolution (Bergmann & Cramer, 1998 ▸; Huotari *et al.*, 2017 ▸; Glatzel *et al.*, 2007 ▸; Mei *et al.*, 2024 ▸). Additionally, the alignment and quality of the multiple crystals are critical considerations. In standard synchrotron experiments utilizing monochromatic X-rays, it is essential to account for the Darwin width of the monochromator crystals and these are summarized in Table 5 ▸. In general, these are accurate for double or quadruple monochromator crystals, but they underestimate the effect on analyser crystals of strain, off-axis aberrations and spherical or cylindrical curvature. Internal calculations by beamline scientists at Diamond Light Source estimate the geometrical broadening for bent crystal analysers with a 1 m vertical geometry to be approximately 898 meV for Mn *K*α and 260 meV for Mn *K*β. These values may vary depending on whether certain critical geometrical parameters are included or omitted.

Perhaps it is preferable to make a similar comparison with literature measurements and modelling estimates, including our data, as in Table 6 ▸. Our comparative analysis of FWHM values for elastic peaks reveals that our findings align with, and in some instances surpass, established trends in the literature (Duan *et al.*, 2017 ▸; Glatzel *et al.*, 2021 ▸; Mei *et al.*, 2024 ▸), as detailed in Table 6 ▸. Specifically, we report raw observed FWHM values of 0.97 eV for the Ge(333) analyser crystal at 5898.6 eV and 0.7 eV for Si(440) at 6490.1 eV, corresponding to Bragg angles of 74.8° and 84.2°, respectively. Perhaps even more importantly, we report spliced FWHMs of 0.56 eV and 0.61 eV, respectively, which should be indicative of the distorted almost cylindrical set of crystal slices of each analyser, including proper curved crystal diffraction, stress and defects in alignment. Hence, these numbers are necessarily significantly larger than the ideal estimates in Table 5 ▸ and are broadly consistent with the estimated analyser bandwidths presented by Glatzel *et al.* (2021 ▸) in Table 6 ▸.

Recent studies, including our own, reflect that elastic peaks sharpen as the Bragg angle approaches 90° (Glatzel *et al.*, 2021 ▸). Duan *et al.* (2017 ▸) documented an elastic peak FWHM of 1.0 eV for Si(440) at similar energies, suggesting comparable resolution, while Glatzel *et al.* (2021 ▸) reported FWHM values of 1.0 eV for Ge(333) at 5851 eV and 0.8 eV for Si(440) across similar energy levels (Glatzel *et al.*, 2021 ▸). This consistency underscores the robustness of the beamline alignment and our results. Future efforts by industry to explore new fabrication techniques for germanium and silicon, along with the integration of alternative materials like quartz and lithium niobate (LiNbO_3_), may enhance the energy resolution and statistics of spectra in appropriate energy ranges. Tables 5 ▸ and 7 ▸ use the Als-Nielsen & McMorrow (2011 ▸) compilation, with form factors incorporated from Chantler (2000 ▸). These are for perfect flat crystals, similar to following Cole & Stemple (1962 ▸) or Hirsch & Ramachandran (1950 ▸), so that these FWHM estimates are unachievable for any real or curved crystal, or for any spherical-like crystal cut into cylindrical slices. Nonetheless, they indicate an ideal attainable with a very different geometry and point towards what might be possible in an HERFD geometry.

We can look at the spatial distribution of the flux diffracted from the crystal analyser, or from a perfect curved crystal slice of the crystal analyser, onto the Rowland circle as a curved crystal detector (like using an image plate or X-ray photographic emulsion), or using a flat plate detector such as the multipixel detectors described herein. In either case, the *Moscurve* software and theory of Chantler can predict the limiting size and position of the diffracted images from a specific ideal source position (Chantler, 1992*a* ▸; Chantler, 1992*b* ▸; Chantler & Deslattes, 1995 ▸). However, that does not address the energy broadening or the raw or spliced FWHM on the detector, which require interpretation of θ_out,surface_, the Bragg angle at the output surface of the analyser; or θ_out_, the apparent Bragg angle output from a reference frame. These are indicated in Table 8 ▸, particularly for proposed future experiments as indicated in previous tables. These estimates are correctly larger than the perfect flat crystal estimate, but note that they do not include misalignment or off-axis errors, nor do they overlap from different cylindrical slices in a spherical-like curved crystal. However, they do suggest that, under optimum conditions, HERFD and XR-HERFD should be able to attain spliced FWHM bandwidths of 0.2–0.4 eV in the future, or possibly better.

Many groups rate the Takagi–Taupin approach as the most accurate, though neglecting a clear definition of the wavefield or ray-tracing path for imaging and, being non-analytic, requiring integration of transport waves, and requiring explicit computation of the strain gradient *etc*. The Chantler approach is also non-analytic and also requires definition of the curvature or strain and stress tensors. The Kato statistical dynamical theory is useful when deformation information is not known but assumed; the Kato and Laue approaches for dynamical diffraction follow or precede Takagi and Taupin and their successors. Unfortunately the above excellent approaches are not analytical and must be numerically solved for each specific optical element – source, crystal, curvature, detector – and most have only been presented for specific geometries. Whilst many have covered perfect crystals or ideal imperfect mosaic crystals and flat or Johann geometries, many of the other real geometries have not been well characterized, and hence some kind of approximate but perhaps analytical estimates are used as above. For the Ewald, Laue, Kato, Takagi and Taupin approaches, an excellent detailed compendium is presented by Authier (2001 ▸).

### 5.1. Illustration: Kapton elastic scattering at low temperatures

**Figure 19:**
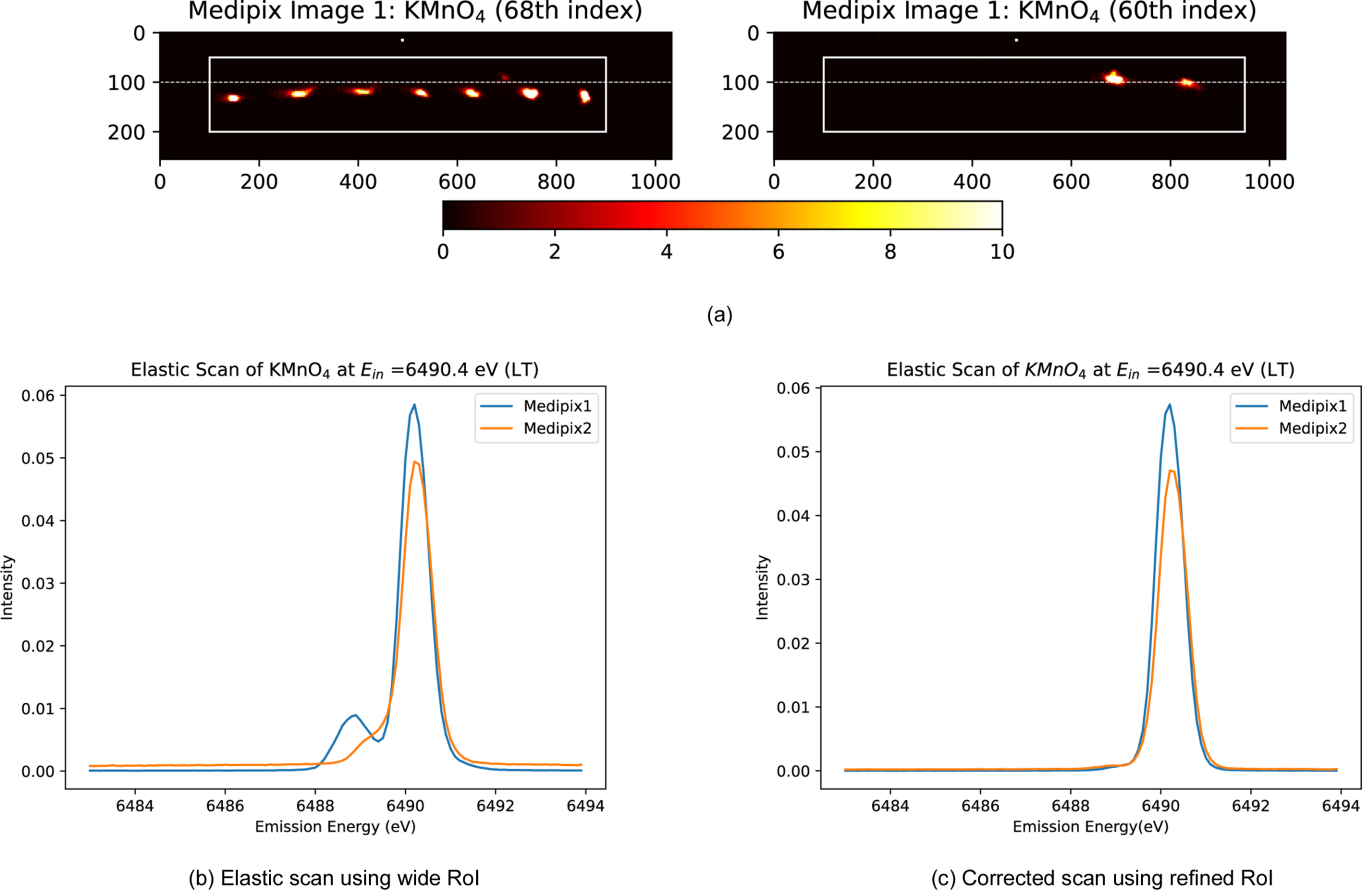
(*a*) First Medipix image of KMnO_4_ at two different indices at low temperature (LT = 11 K). The left-hand portion displays the image of seven crystals of the chosen sample, while the right-hand portion shows the Kapton fringe appearing at completely different indices. Here, the index refers to a consecutive sequence of emission energies. (*b*) Elastic scan of KMnO_4_ at an incident energy (*E*_in_) of 6490.4 eV. The entire white-marked rectangular area is selected as the region of interest (RoI). The result depicts an additional bump just before the main peak, which represents the influence of the Kapton tape from our experimental setup. (*c*) Corrected elastic scan utilizing our standard approach for RoI selection, as discussed in this paper, effectively removing the Kapton influence.

During Experiment 4 on the I20-Scanning beamline, the cryostat maintained temperature stability across a range of 10 K to 250 K during the measurement of various manganese samples. However, elastic scattering from the Kapton windows was consistently observed in the Medipix images near the elastic peaks of the samples – in the lower left of the second image and the upper right of the first – due to the inverted orientations of the detectors. These uninvited fringes can badly distort the spectrum, but they can be isolated if properly identified. Usually, these unwanted peaks arise from the beam scattering elastically from the Kapton window, rather than from the sample, and hence from different positions on the Rowland circle compared with the sample surface, producing separated peaks as in Fig. 19 ▸. This unwanted additional substructure can be isolated spatially and eliminated using our standard approach (Section 2.2). Results are presented in Fig. 19 ▸(*c*). Additionally, this requires systematically examining the Medipix images at each index to identify and isolate these Kapton peaks accurately. KMnO_4_ is illustrated as an example with a similar alignment to the Mn foil samples.

### 5.2. XAFS scans using the 14-crystal spectrometer

**Figure 20:**
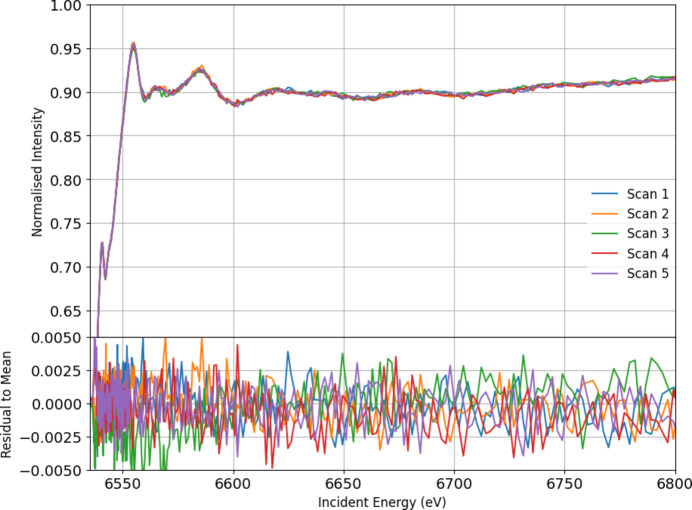
Five repeated XAFS scans of Mn foil at room temperature using the 14-crystal spectrometer at emission energy 6490.4 eV. The repeated scans clearly depict the consistency and precision of our setup and energy calibration. Residuals, calculated by subtracting each individual scan from the overall mean, are also presented at the bottom.

Fig. 20 ▸ presents the key aspect of our XAFS measurements conducted at room temperature using the 14-crystal spectrometer. It shows the results of five repeated XAFS scans of manganese foil, demonstrating the high precision and consistency of our experimental setup. Each scan, performed at an emission energy of 6490.4 eV, exhibits minimal variance and emphasizes the spectrometer’s capability in resolving subtle spectral differences.

## 6. Conclusions

This article outlines key features of the I20-Scanning spectrometer and demonstrates the spatial resolution provided by the detector, which utilizes each pixel area to optimize statistics, counts and structural detail for splicing. Key improvements include reduced spectral broadening and increased signal intensity. The splicing techniques have achieved up to a 46% enhancement in resolution. This approach enables more critical comparisons with theory, especially for high-resolution spectra and XR-HERFD spectra. These innovations provide new opportunities in X-ray spectroscopy and will hopefully aid understanding of material properties. We have discussed different empirical and semi-empirical estimates of Darwin FWHM bandwidths in energy with contributions from curvature defects, spherical or cylindrical crystal strain and off-axis or off-Rowland circle misalignments, and have also looked at ideal estimates for ideal perfect flat crystals, and for realistic but ideal cylindrical curved crystals, as templates for potential improvement in the future.

## Figures and Tables

**Figure 5:**
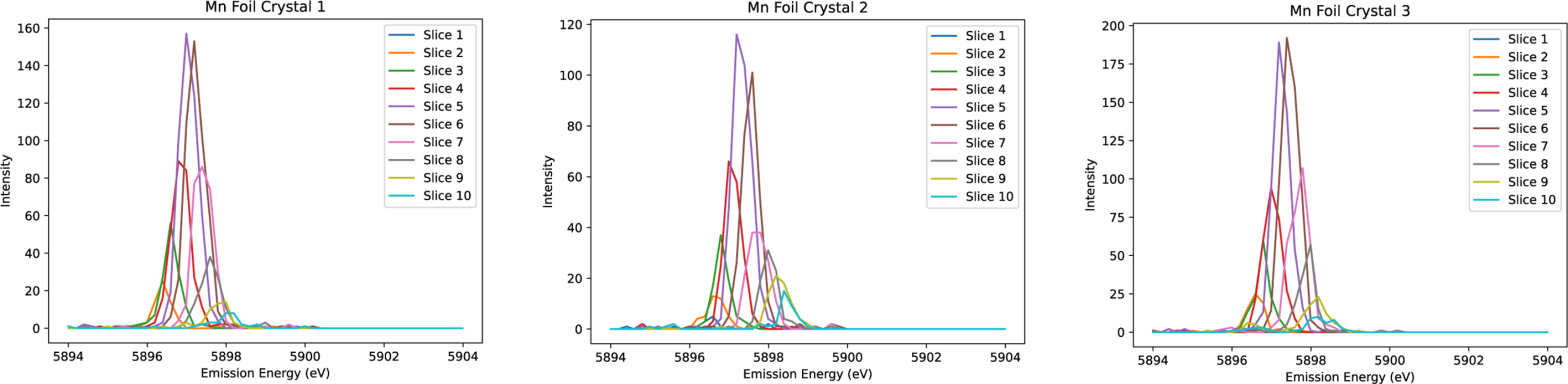
Elastic scans of manganese foil from Experiment 1 at room temperature. Each crystal is split into ten slices, each of which clearly exhibits some shift in the peak emission energy.

**Figure 12:**
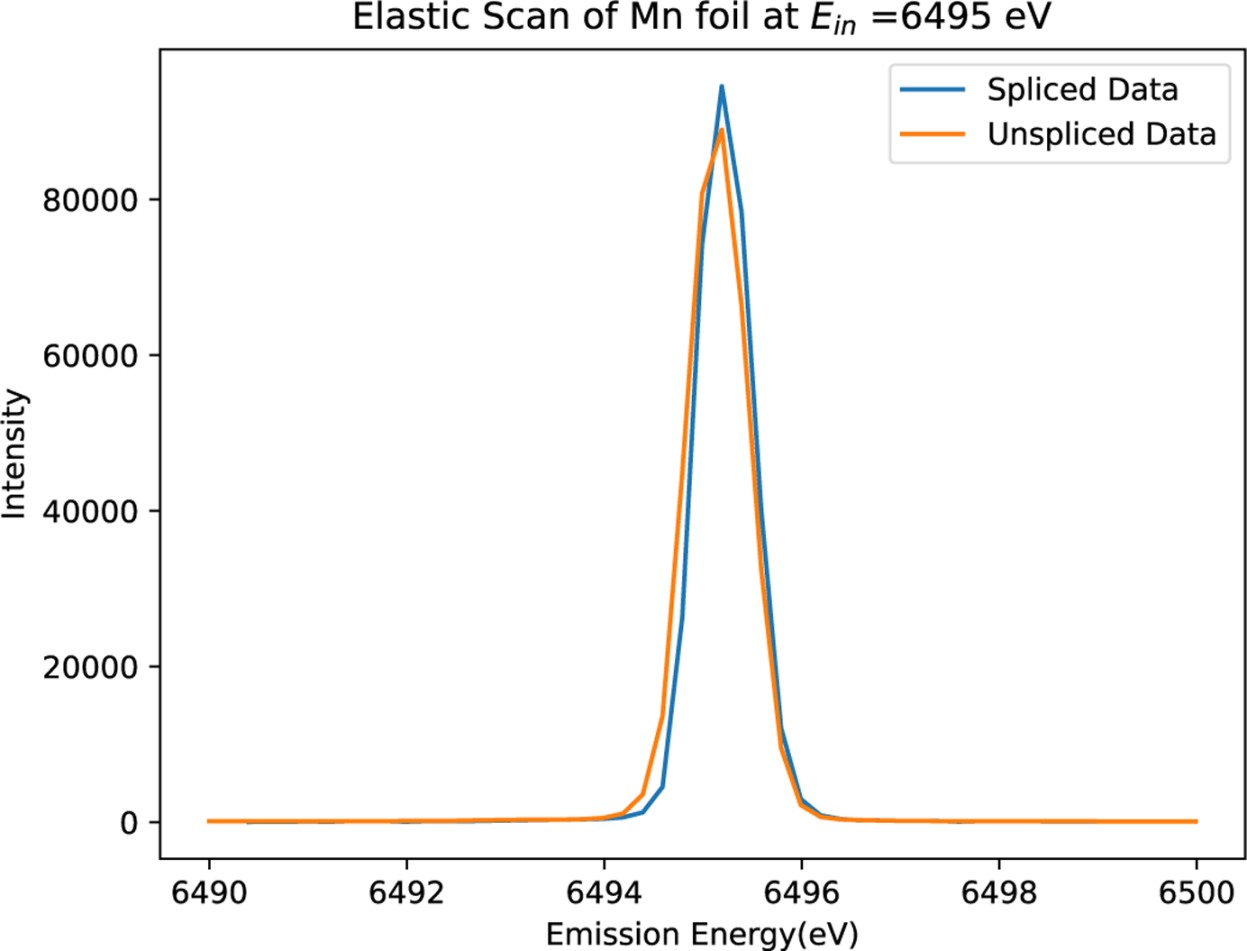
Spliced (processed, blue) and unspliced (raw, orange) spectra of the Mn foil using the 14-crystal analyser (Experiment 3). The signal intensity is increased compared with Experiments 1 and 2. Splicing has enhanced the peak counts. Eight working crystals are included in the plot.

**Figure 17:**
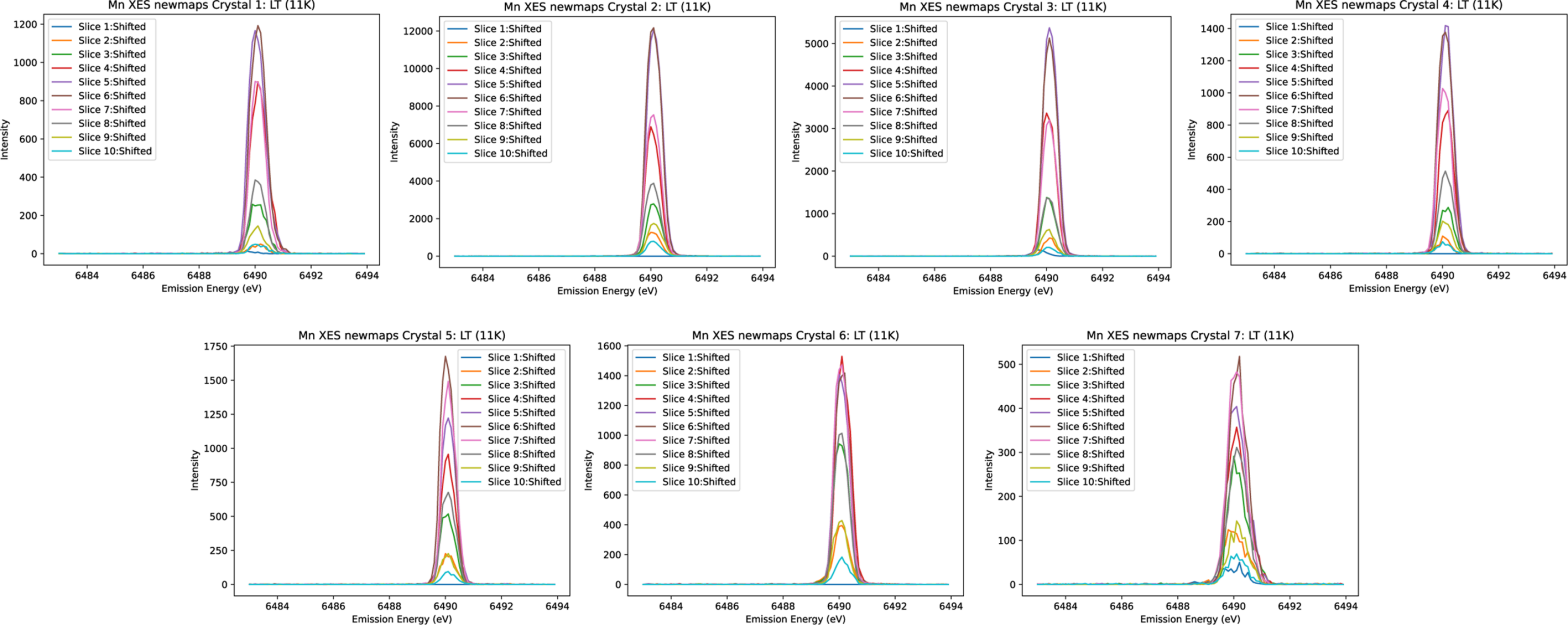
Elastic scans of seven crystals displaying spectra of ten slices as shown in Fig. 16 ▸, following a shift relative to the reference spectra.

**Table 1:** A comparative analysis of the results from Experiments 1 and 2, illustrating the effects of splicing on measurement parameters

	Experiment 1			Experiment 2		
	Unspliced data	Spliced data	% change	Unspliced data	Spliced data	% change
Peak energy (eV)	5897.19	5897.19	0	5899.39	5899.39	0
Peak count	988	1809	83.1	811	1290	59.1
FWHM	1.039	0.556	46.5	0.977	0.572	41.5

**Table 2:** Improvements observed with the 14-crystal spectrometer and splicing

	Peak energy (eV)	FWHM	Peak count
Unspliced data	6490.1	0.70133	158285.0
Spliced data	6490.1	0.6157	174332.2
% change	0	12.2	10.1

**Table 3:** Contributions to the final resolution function for a spherical crystal (Huotari *et al.*, 2017 ▸)

Contribution	δ*E*, eV at 9.9 keV variation
Strain aberration	0.1–2.0
Off-Rowland circle defect *z*	0.11–1.4
Incident bandwidth from monochromator (Shvyd’ko, 2004 ▸)	0.25–1.78

**Table 4:** Contributions to the final resolution function, Johann mounting (Huotari *et al.*, 2005 ▸), for an Si(555) backscatter which removes the dominant 1.4 eV Si(111) typical analyser width

Contribution	δ*E*, meV at 9.9 keV
Analyser crystal Darwin width	15
Source size	10
Pixel size	7
Johann aberration	<1
Total spectrometer	23
Incident bandwidth from monochromator	15
Total resolution estimate	27

**Table 5:** Calculated Darwin widths and Bragg angles for monochromator and analyser crystals at selected energies for perfect crystal Bragg reflections using dynamical diffraction theory (Takagi, 1962 ▸; Taupin, 1964 ▸) These values were computed using the X-ray Data Bank (XrayDB) by the International X-ray Absorption Society (IXAS) and Matthew Newville, incorporating Chantler atomic form factors, as described by Als-Nielsen & McMorrow (2011 ▸). The sixth column presents widths from the Takagi–Taupin module using X-ray optics utilities (*XOP*) (Dejus & Sanchez del Rio, 1996 ▸). Broadening arising from stress in the lattice planes due to bending of the crystal will further increase the width.

				Energy Darwin width (eV)		
	Reflection	Energy (eV)	Bragg angle (°)	XrayDB	*XOP*	Angular Darwin width (µrad)
Monochromator	Si(111)	5898.6	19.583	0.620	**	37.409
	Si(111)	6490.1	17.735	0.714	**	35.223
Analyser crystal	Ge(333)	5898.6	74.842	0.114	0.104	71.431
	Si(440)	6490.1	84.222	0.066	0.100	101.837

** No data given.

**Table 6:** Comparison of monochromator and analyser crystal configurations: measured experimental FWHM values for elastic scans near Mn *K*α and *K*β emission energies This table summarises and compares the most relevant work from different synchrotrons with our findings.

Reference	Energy (eV)	No. of analyser crystals	Monochromator crystal reflection	Analyser crystal reflection	Analyser crystal Bragg angle (°)	Elastic peak FWHM (normal data) (eV)	Elastic peak FWHM (spliced data) (eV)	Estimated analyser bandwidth (eV)
This work	5898.6	3	Si(111)	Ge(333)	74.8	0.97	0.56	**
	6490.1	14	Si(111)	Si(440)	84.2	0.7	0.61	**
Duan *et al.* (2017 ▸)	6490.1	3	Si(111)	Si(440)	84.19	1	**	**
Glatzel *et al.* (2021 ▸)	5851	5	Si(111)	Ge(333)	76.70	1.0	**	0.6
	6492	5	Si(311)	Ge(440)	72.70	0.8	**	0.7
	6500	5	Si(111)	Si(440)	84.10	1.0	**	0.4
Mei *et al.* (2024 ▸)	6499.5	7	Si(111)	Si(440)	81.52	0.8	**	**

** No data given.

**Table 7:** Calculated Darwin widths and Bragg angles for monochromator and analyser crystals at selected energies for (our) future experiments

	Reflection	Energy (eV)	Bragg angle (°)	Energy Darwin width (eV)	Angular Darwin width (µrad)
Monochromator	Si(111)	4091 (Sc *K*α_1_)	28.899	0.298	40.270
	Si(111)	8638 (Zn *K*α_1_)	13.231	1.037	28.239
Analyser crystal	Si(642)	8638 (Zn *K*α_1_)	81.449	0.037	28.768
	Ge(555)	9572 (Zn *K*β)	82.448	0.041	32.463
	Si(311)	4091 (Sc *K*α_1_)	67.729	0.085	51.073
	Ge(400)	4462 (Sc *K*β)	79.186	0.268	315.537

**Table 8:** Tabulation of FWHM for finite curved (imperfect) crystals of Si and Ge at different Miller indices from Chantler *Moscurve* theory (Chantler, 1992*a* ▸; Chantler, 1992*b* ▸; Chantler & Deslattes, 1995 ▸) for possible new experiments

Crystal	Reflection	Energy (eV)	Bragg angle (radians)	FWHM θ_out,surface_ (eV)	FWHM θ_out_ (eV)
Si	311	4091	1.180	0.126	0.062
	642	8638	1.421	0.330	0.072
Ge	400	4462	1.38	0.104	0.091
	555	9572	1.438	0.103	0.129

## Data Availability

The data supporting the findings of this study are available upon request.
